# Experimental and Theoretical Insights into Nanoscale AFM-IR Imaging of Complex Heterogeneous Structures

**DOI:** 10.1021/acs.analchem.5c04707

**Published:** 2025-09-18

**Authors:** Yide Zhang, Ufuk Yilmaz, Artem S Vorobev, Simone Iadanza, Liam O’Faolain, Bernhard Lendl, Georg Ramer

**Affiliations:** † 27259 Institute of Chemical Technologies and Analytics, TU Wien, Vienna 1060, Austria; ‡ Centre for Advanced Photonics and Process Analysis, 587895 Munster Technological University, Cork T12P928, Ireland; § Tyndall National Institute, Cork T12R5CP, Ireland; ∥ 28498 Laboratory of Nano and Quantum Technologies Paul Scherrer-Institut, Villigen 5232, Switzerland; ⊥ Ecole Polytechnique Federale de Lausanne, Lausanne 1015, Switzerland; # Christian Doppler Laboratory for Advanced Mid-Infrared Laser Spectroscopy in (Bio-)process Analytics, TU Wien, Vienna 1060, Austria

## Abstract

Nanoscale chemical imaging enabled by atomic force microscopy-infrared spectroscopy (AFM-IR) provides valuable insights into the complex structures and chemical compositions of materials and biological samples. While AFM-IR has been applied to subsurface imaging, the underlying mechanisms, particularly in nonplanar geometries and complex heterogeneous structures, remain underexplored. This study presents a theoretical analysis and experimental validation of AFM-IR for imaging subsurface features within organic multilayer structures, uncovering how image broadening depends on whether the excitation occurs in the subsurface or the covering layer. An analytical model based on the sample geometry demonstrates that the lateral size of the absorber significantly impacts both the signal intensity and spatial resolution in AFM-IR chemical imaging. These findings are experimentally validated, and a more representative finite element method (FEM) model was subsequently created, resulting in strong agreement with the experimental data. The model reveals how irregular structures directly impact photothermal expansion, providing an explanation for the distinct image broadening observed with infrared excitation of different layers. Additionally, a linear relationship is observed between feature size, chemical images, and AFM-IR signal intensity. These findings contribute significantly to the understanding of the AFM-IR signal, providing insights into resolution and sensitivity, paving the way for more advanced nanoscale chemical imaging capabilities.

## Graphical abstract

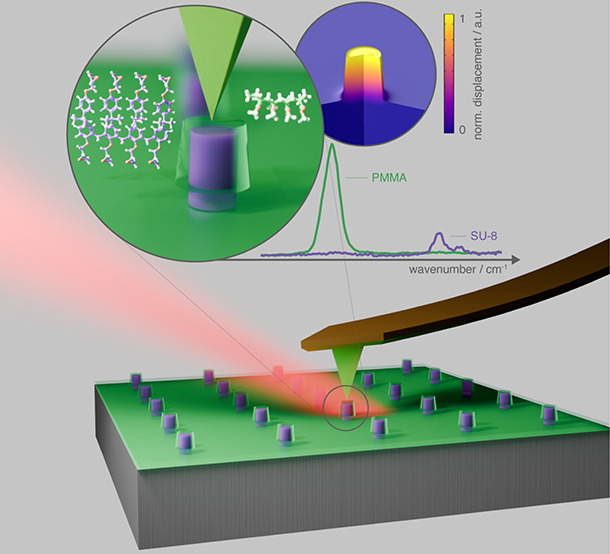

## 1 Introduction

Achieving nanoscale chemical imaging with high spatial resolution remains a challenge, particularly for subsurface structures where traditional techniques struggle to provide accurate depth information. One technique that offers nanoscale chemical sensitivity is atomic force microscopy-infrared spectroscopy (AFM-IR), which combines the spatial resolution of atomic force microscopy with the chemical specificity of infrared absorption. It enables subdiffraction-limited chemical imaging with lateral resolution typically ranging from 5 to 20 nm, depending on the configuration.^1–4^

AFM-IR is known to detect signals from structures buried up to micrometer depths, offering chemical information even in layered or inhomogeneous samples.^5–7^ However, the mechanisms governing this subsurface sensitivity remain poorly understood. Only a limited number of empirical and theoretical studies have begun to investigate the origins, limits, and interpretability of such depth-dependent signals.^8–10^

The working principle of AFM-IR lies in detection of thermal expansion of the sample area beneath the cantilever tip after the absorption of focused, pulsed IR radiation. A sequence of laser pulses induces a modulated temperature change in the sample, generating photothermal and photoacoustic effects due to radiation absorption.^11^ This results in a rapid thermal expansion and a height change of the sample surface, which then induces an oscillatory motion in the cantilever, detected through the cantilever deflection signal. The extent of this deflection is directly proportional to both the wavelength-dependent absorption coefficient and the thermal expansion coefficient of the sample.^12^ This enables AFM-IR to provide images based on local infrared absorption, i.e., chemical imaging.

While the cantilever tip scans the sample surface only, the AFM-IR signal is not just from the topmost part of the sample. Several studies have investigated depth sensitivity by optimizing laser parameters. For instance, Prine et al. demonstrated tunable depth sensitivity in bilayer polymer films by controlling laser energy and pulse frequency, while Jakob et al.^9^ explored how signal intensity varies with sample thickness. Dazzi et al. established an empirical relationship between probing depth and laser repetition rate. These studies have highlighted the value of AFM-IR as a nondestructive technique for observing subsurface features at the nanoscale.

However, these prior studies focused on simplified layered structures with uniform interfaces, where depth profiling could be predicted with well-controlled conditions. In real-world applications, many materials exhibit complex three-dimensional geometries, irregular sample topographies, and lateral heterogeneities that affect AFM-IR signal generation and spatial resolution. The mechanisms governing AFM-IR response in such systems remain largely unexplored.

The need for nondestructive subsurface imaging^13^ is critical for understanding defects and dislocation layers beneath the surface^14^ particularly in micro- and nanodevice manufacturing for detecting buried defects^15^ energy science for probing subsurface morphologies that influence charge transport and stability^16^ and semiconductors for imaging nanoscale buried interfaces essential for device optimization.^17^

While AFM struggles with depth resolution^18^ other infrared-based scanning probe microscopy techniques, such as scattering-type scanning near-field optical microscopy (s-SNOM)^19,20^ and nano-FTIR spectroscopy^21^ have emerged as promising alternatives for surface and subsurface measurements. These techniques offer spatial resolution in the order of the tip radius, typically around 25 nm, providing valuable insights into nanotomography and enabling simultaneous chemical composition and topography measurements. They also allow depth probing up to 100 nm.^21,22^ However, these measurements were limited to simple structures, such as thin-film or stair-shaped samples. Furthermore, they did not fully account for the sample’s geometry and the influence of sample size has been largely overlooked.

Our recent work demonstrated the depth and size dependence of spherical absorbers on signal intensity and spatial resolution in AFM-IR.^23^ However, knowledge about the absorber position in this work was only indirect and the sample surface was required to be flat. Further experimental validation and a nontrivial extension of the model are needed to understand the AFM-IR signal of subsurface absorbers in complex geometries.

In this study, we improve upon our previous work and the state of the art in several crucial ways: We now use nanofabrication to create our samples. This yields well controlled complex heterogeneous structures. We thus have direct knowledge about absorber size, shape and position and the sample surface no longer is flat, enabling us to explore the effect of surface topography on the AFM-IR signal. Nanofabrication also means that sample dimensions can easily be changed through nanofabrication, which allows verification of the models across a large parameter space. With these controlled structures, we establish a direct correlation between experimental observations and theoretical predictions. Furthermore, we experimentally confirm a linear relationship between absorber size and AFM-IR signal broadening, providing new insights into spatial resolution and signal interpretation in AFM-IR imaging.

## 2 Results and Discussion

Samples were fabricated on silicon substrates with 1 μm-high SU-8 (a high contrast, epoxy-based electron beam resist) structures using electron-beam lithography (EBL). The sample structures are depicted in step c of Figure 1A and are referred to as “SU-8” in figures and throughout this work. The patterned SU-8 was then coated with a 185 nm-thick PMMA layer, illustrated in step d of Figure 1A. This bilayer sample is referred to as “SU-8 & PMMA” throughout the manuscript. The resulting patterns are illustrated in Figure 1B.

**Figure 1:**
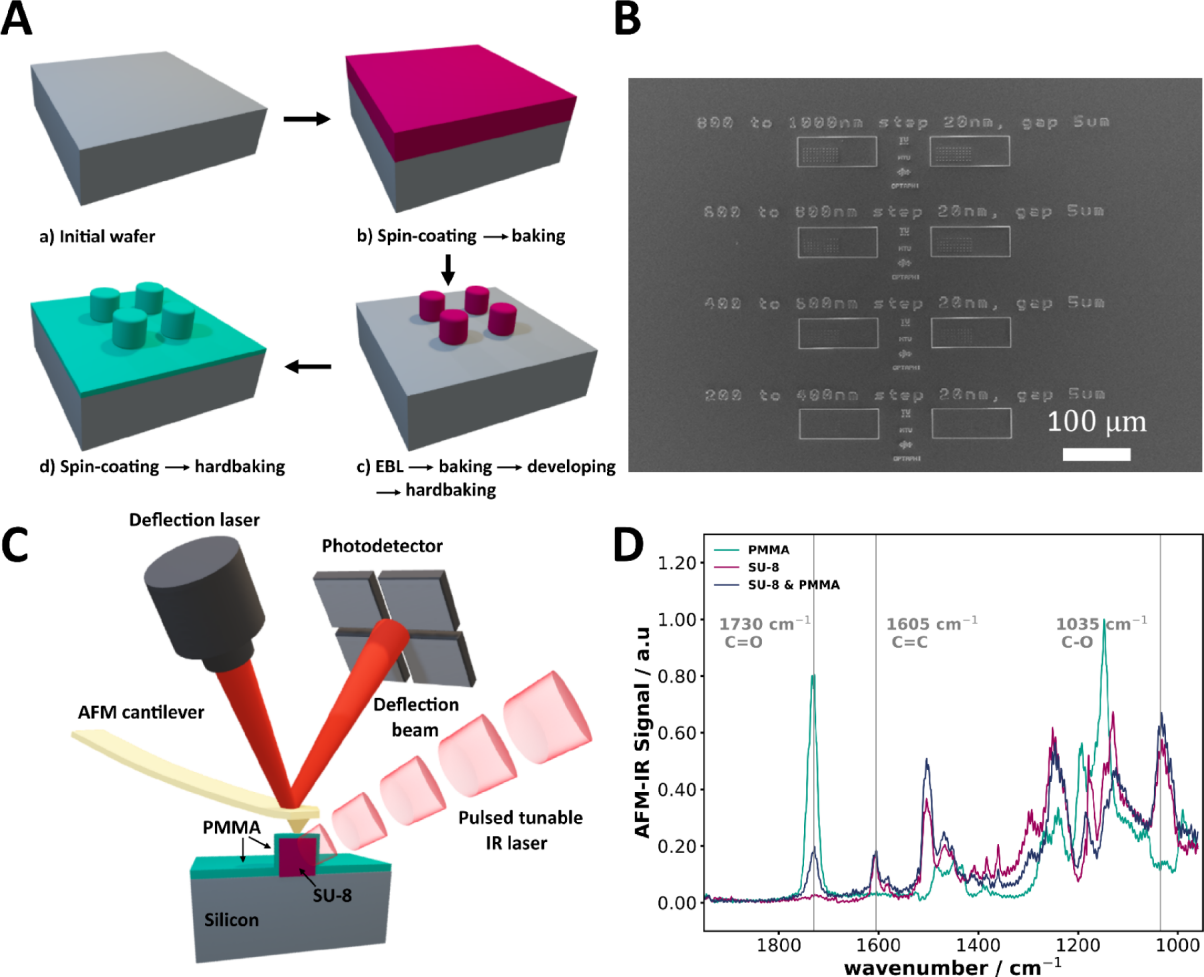
(A) Fabrication steps for the sample (details are discussed in the Methods section). The SU-8 layer is depicted in purple, while the PMMA layer is depicted in turquoise. (B) SEM image of the fabricated sample, featuring a 1 μm thick layer of SU-8 on a silicon substrate, encased by a 185 nm layer of PMMA. The pillars have diameters ranging from 480 to 1000 nm, with steps of 20 nm, along with various logo-shaped structures on the same chip. (C) Schematic of the AFM-IR setup. The pulsed excitation IR beam (red) illuminates the sample, inducing local photothermal expansion. The AFM cantilever (gold) detects the resulting surface deformation, which is measured by the detector. The sample consists of an SU-8 layer (purple) on a silicon substrate, covered by a PMMA layer (turquoise). (D) AFM-IR spectra obtained from the SU-8 and SU-8 & PMMA samples, respectively.

In the experiments, we employed tapping-mode AFM-IR^4,11,24–26^ which utilizes the mechanical resonance of the cantilever and heterodyne mixing of the cantilever tapping motion and surface expansion to enhance signal detection. Unlike other modes, the resonance frequency is minimally influenced by variations in the sample’s mechanical properties.^27^ Moreover, this approach delivers exceptional spatial resolution, achieving lateral resolutions as fine as 10 nm.^4^ The AFM-IR spectrum of SU-8 was obtained from the SU-8 sample, while the spectrum of PMMA was obtained from the SU-8 & PMMA sample, specifically from the surrounding area of pillars. SU-8 is characterized by the presence of the C=C stretching vibration near 1605 cm^–1^ and the C–O stretching vibration around 1035 cm^–1^.^28^ In contrast, PMMA exhibits a distinct absorption band at 1730 cm^–1^, attributed to the C=O vibrational mode of the ester moiety. Positioning the cantilever tip atop of the nanopillar, produced a combination from both SU-8 and PMMA spectra, shown as the dark-blue curve in Figure 1D.

To investigate the dependence of AFM-IR signal intensity and spatial resolution on the size of absorbers, we designed and fabricated nanopillars with identical height but varying diameters, ranging from 480 to 1000 nm, as shown in Figure 1B. AFM-IR experiments were performed at laser wavelengths corresponding to the absorption bands of the underlying SU-8 and the covering PMMA layers. The same AFM cantilever was used throughout all pillar measurements to minimize any mechanical variability between probes that could affect the signal intensity. Topography and chemical images were collected at each wavenumber over a 10 μm by 10 μm area containing four pillars (a single pillar measurement is presented in Figure S2). For more measurement details, see the Methods section.

Figure 2A shows an SEM image of four pillars. Pillars aligned in the same column in the top and bottom rows are identical in size. The pillars in the right column have a diameter that is 20 nm larger than those in the left column. SEM measurements provided a rough estimate of pillar dimensions, the fidelity of the fabrication process. To map the C=C stretch band of SU-8, chemical images at 1605 cm^–1^ were recorded (Figure 2B). Areas of strong absorption in Figure 2B correspond to the higher (white) regions in the topography image (Figure 2C). By tuning the laser to 1730 cm^–1^ and exciting PMMA’s C=O vibrational mode, we recorded a chemical image of the same area (Figure 2D). While the distinct shape of the pillars remains clearly visible, the highest IR signal is observed in the surrounding area rather than at the pillars themselves.

**Figure 2:**
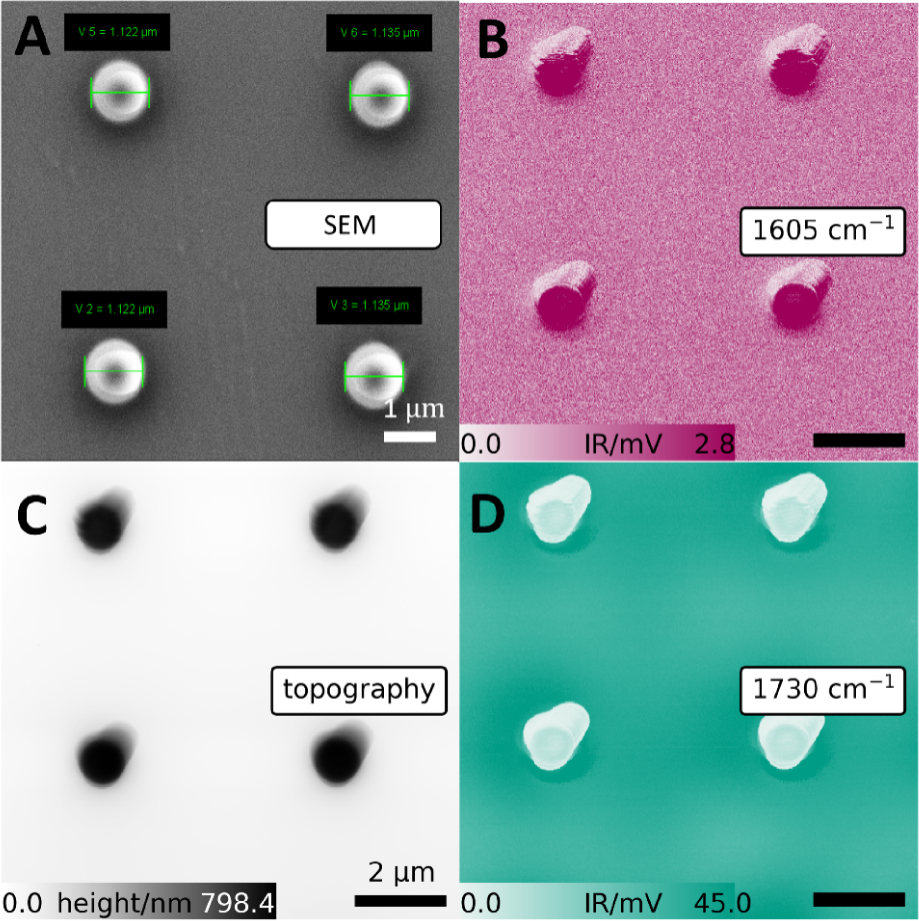
(A) SEM image of fabricated SU-8 pillars covered by PMMA. The first column shows SU-8 pillars with a designed diameter of 800 nm, and the second column 820 nm. SEM confirms final diameters of 1122 and 1135 nm, respectively, including PMMA thickness. Note that the turquoise markings include the thickness of the covering PMMA layer. (B) AFM-IR chemical map at 1605 cm^–1^. (C) AFM topography image. (D) AFM-IR chemical map at 1730 cm^–1^. The scale bar is consistent across panels B, C, and D.

Based on the topographic and chemical imaging measurements of the nanopillars, we analyzed the dependence of AFM-IR signal intensity and resolution on the absorber size. To achieve this, we generated cross-sectional profiles through the center of each nanopillar, measured the fwhm, and averaged the IR signal in the integrated area for each, as shown in Figure S3. Figure S4A shows the cross-sectional profiles of the pillars in the first row of Figure 2B and C, while Figure S4B presents the profiles from the first row of Figure 2C and D.

To gain deeper insight into the physical origins of the measured AFM-IR signal and account for geometric and material effects, we developed an analytical model based on the two-dimensional transient heat conduction and thermoelastic equations in cylindrical coordinates. Specifically, we modeled laser heating as a time-dependent cylindrical volumetric heat source *g*(*r,z,t*), where *r* and *z* represent any location in the domain. Heat is generated internally throughout the solid absorber at a rate of *g*(*r,z,t*) per unit volume. Thermal conduction in cylindrical coordinates was described using Fourier’s law^29^

1 $$\frac{\partial^{2}T\left(r,z,t\right)}{\partial r^{2}}+\frac{1}{r}\frac{\partial T\left(r,z,t\right)}{\partial r}+\frac{\partial^{2}T\left(r,z,t\right)}{\partial z^{2}}+\frac{g\left(r,z,t\right)}{\kappa }=\frac{1}{\alpha }\frac{\partial T\left(r,z,t\right)}{\partial t}$$

where *κ* is the thermal conductivity and α is the thermal diffusivity. In the equilibrium state, the solution of the Navier’s equations of thermoelasticity^30^ without external force in the cylindrical coordinates can be expressed by the Boussinesq harmonic functions:

2 $$u_{z}=\frac{\partial \Phi }{\partial z}+\frac{\partial \varphi }{\partial z}+z\frac{\partial \psi }{\partial z}-\left(3-4v\right)\psi$$

3 $$u_{r}=\frac{\partial \Phi }{\partial r}+\frac{2}{\partial r}\frac{\partial \varphi }{\partial r}+z\frac{\partial \psi }{\partial \theta }+z\frac{\partial \psi }{\partial r}$$

where *v* is Poisson’s ratio, Goodier’s thermoelastic displacement potential Φ and Boussinesq harmonic functions φ and ψ must satisfy governing equations,^30^ as shown in eqs S58, S60 and S61.

In this study, tapping-mode AFM-IR is utilized, ensuring that the cantilever tip remains perpendicular to the sample surface. This configuration effectively eliminates lateral tip–sample forces.^31^ Consequently, lateral forces are negligible, and only the vertical displacement *u*_*z*_ is considered as the primary factor contributing to surface deformation.

Assuming that both the buried and covering layers are perfect cylindrical shapes with vertical sidewalls, the surface displacement can be derived and simplified as follows (see Supplementary Section S2):

4 $$u_{z}\left(r,t\right)=\overset{\infty }{\underset{n=0}{\sum }}\overset{\infty }{\underset{m=0}{\sum }}\frac{2\left(1+v\right)A\left(\beta_{m},\eta_{n}\right)\alpha_{z}}{\beta_{m}}\left(\frac{\eta_{n}^{2}}{\beta_{m}^{2}+\eta_{n}^{2}}+1\right)\underset{\mathrm{spatial}}{\underset{︸}{J_{0}(\beta_{m}r)}}\underset{\mathrm{temporal}}{\underset{︸}{\tau \mathrm{(t)}}}$$

Here, β_*m*_ and η_*n*_ represent eigenvalues determined by the boundary conditions. However, two boundary conditions—thermal insulation at *r* =*R*_mat_ and *z* =*H*_mat_—are not included in the model. Incorporating these conditions would prevent the use of separation of variables. Nevertheless, comparisons with FEM simulations confirm that this simplification has minimal impact on the results. The term *A*(*β*_m_,*η*_*n*_) is the modal amplitude (refer to Supplementary Section S1 eq S52).

5 $$A\left(\beta_{m},\eta_{n}\right)\propto \frac{g_{V}R_{\mathrm{abs}}J_{1}\left(\beta_{m}R_{\mathrm{abs}}\right)}{\kappa \lambda_{mn}^{2}\beta_{m}}$$

This term primarily depends on the product of absorber size *R*_abs_ and solutions of first order Bessel functions *J*_1_(*β_m_R*_abs_), volumetric heat source *g*_*V*_ (the product of the optical absorption coefficient and optical fluence), and the eigenvalue solutions.

From eqs 4 and 5, it is evident that the amplitude of surface displacement depends linearly on the optical absorption coefficient and thermal expansion coefficient α_*z*_, aligning with previous findings.^12,23^ Additionally, it is inversely proportional to the thermal conductivity κ.

The AFM-IR signal also exhibits a distinct dependence on absorber size. As shown in Figure 3A, the average signal scales with absorber volume for sizes below approximately 840 nm, while beyond this threshold, it transitions to a linear relationship with absorber diameter. Within the absorber diameter range of the fabricated samples, the averaged signal follows a linear trend, as highlighted in the inset of Figure 3A.

**Figure 3:**
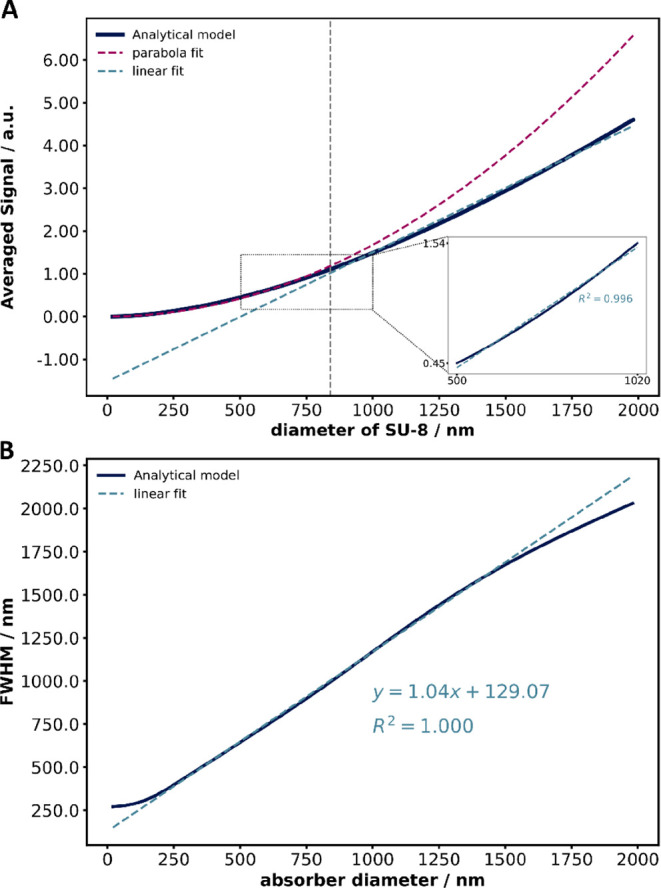
(A) The simulated averaged signal and (B) the full width at half-maximum (fwhm) of the surface displacement profiles are plotted as functions of the SU-8 diameter, based on the analytical model. The linear fit in (B) was performed for SU-8 diameters ranging from 200 to 1500 nm. The analytical simulations were conducted at a laser repetition rate of 710 kHz and a laser pulse width of 220 ns, matching the experimental conditions.

Moreover, the fwhm of the surface displacement profile closely follows the absorber diameter, increasing linearly from 200 to 1500 nm. For diameters below 200 nm, this trend deviates, reaching a minimum of 270 nm. Beyond 1500 nm, the fwhm tends to match the absorber diameter, as shown in Figure 3B. These findings highlight the strong dependence of AFM-IR spatial resolution on absorber size.

To accurately model the nanopillar structure while reflecting its real-world fabricated characteristics, we analyzed an SU-8 pillar with 800 nm diameter, by measuring its height from topography images of both the SU-8 and SU-8 & PMMA samples. Cross-sectional profiles through the pillar centers (Figure 4A) show that the sidewalls are not perfectly vertical but exhibit a tilt, particularly in the SU-8 & PMMA sample. This effect cannot be accurately modeled using an analytical approach. Therefore, we developed a more realistic FEM model to compare the results with experimental data. Detailed descriptions of the simulations are provided in the Supplementary Section S4. In our model, we assume tilted sidewalls with flat surfaces for both the SU-8 and PMMA covering layers, as depicted in Figure 4B and C. A cylindrical coordinate system was used to achieve an axi-symmetric, quasi-3D representation. This approach is more intuitive than a full 3D model, while still retaining generality, and offers significantly lower computational cost, making it more suitable for comparison between the analytical model and numerical model.

**Figure 4:**
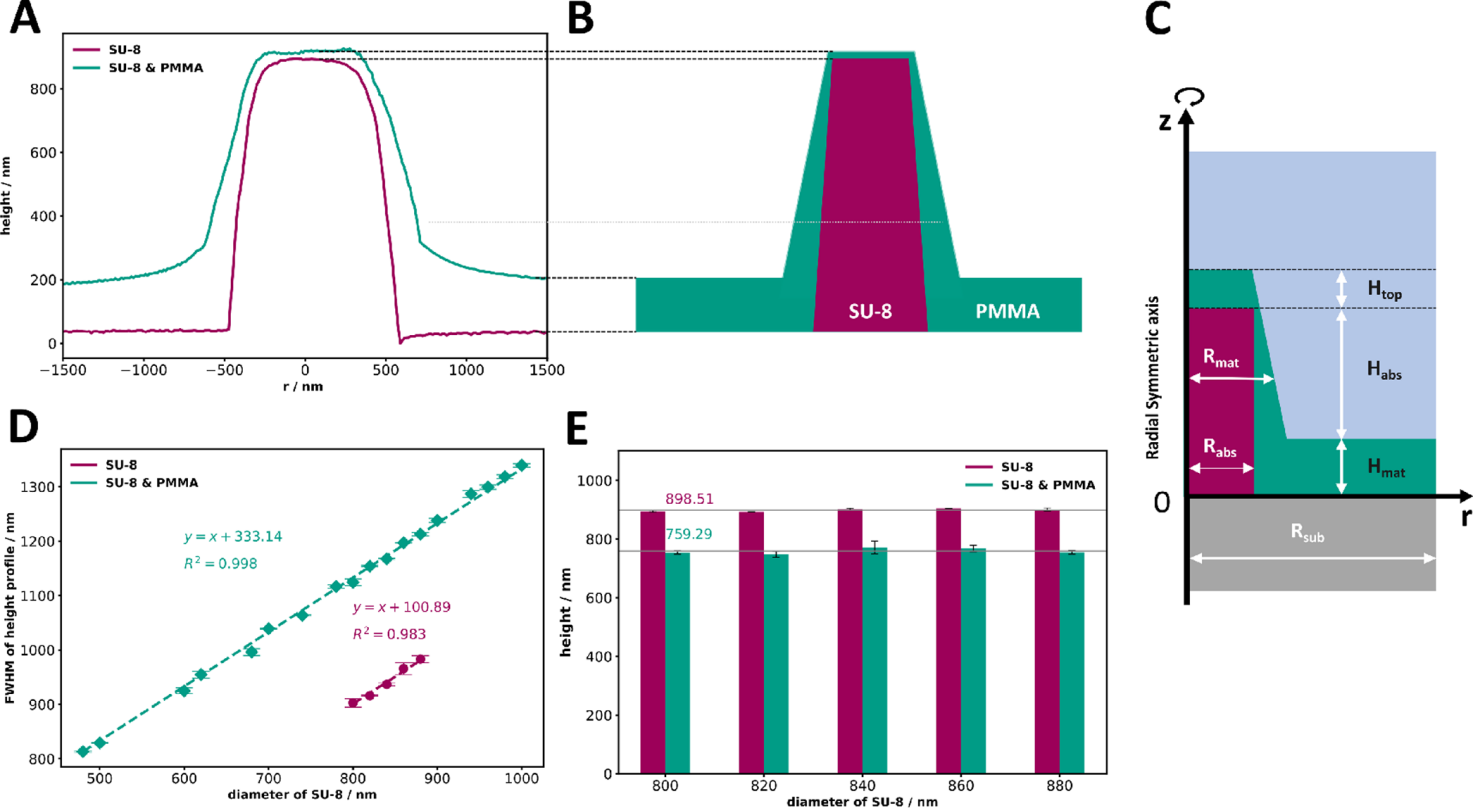
(A) Cross-sectional height profiles of a nanopillar with an 800 nm diameter were obtained for SU-8 and SU-8 & PMMA samples. (B) Schematic representation of the modeled nanopillar. In the graph, the dashed line indicates the reference height from panel A, reflecting the geometry of the SU-8 and PMMA layers. (C) Cylindrically symmetric system composed of a single cylindrical absorber surrounded by a matrix, deposited on a nonabsorbing substrate. (D) fwhm of the cross-sectional height profiles of the SU-8 and SU-8 & PMMA samples. Error bars indicate the maximum deviation from the mean values. (E) Height of each nanopillar at different designed diameter of SU-8. Note that the SU-8 & PMMA structures appear shorter than the SU-8 only structures due to a change in reference height: in the SU-8 only case, height is measured from the silicon substrate, while in the SU-8 & PMMA case, it is measured from the surrounding PMMA surface, which is thicker on the substrate and thinner over the SU-8 features.

Furthermore, to ensure the accuracy of the simulations, we conducted measurements on the SU-8 sample to determine the height and fwhm of each nanopillar (see Figure S4). The fwhm of each nanopillar was determined from its height cross-sectional profiles, revealing a linear relationship with the diameter of SU-8 for both SU-8 and SU-8 & PMMA samples, as shown in Figure 4D, confirming consistency between the design and the fabrication process. From the linear fit, the sidewall thickness was calculated as the difference of the radius of the PMMA layer (*R*_*mat*_) and SU-8 pillar (*R*_*abs*_). Using the measured values, the thickness is given by *R_mat_* – *R_abs_* = 333.14 nm/2 – 100.89 nm/2 = 116.1 nm.

The height of examined nanopillars exhibited only minor variations within each sample, as depicted in Figure 4E. Notably, the average height of the SU-8 sample is greater than that of the SU-8 & PMMA sample, suggesting an inhomogeneous coverage of PMMA on the substrates and SU-8 structures. Using the measured average heights of selected pillars, we calculated *H*_*abs*_ + *H*_*mat*_ = 898.5 nm, *Htop* + *H_abs_* = 759.3 nm, where *H*_*abs*_ the height of absorber, *H*_*mat*_ the PMMA layer thickness on the substrate, *H*_*top*_ the PMMA layer thickness on the SU-8 nanopillar, as as illustrated in Figure 4C. The PMMA layer thickness on substrate was determined by height measurements at six locations using a profilometer, yielding an average thickness of *H_mat_* = 171.9 nm. From this, the PMMA layer thickness on SU-8 nanopillar was calculated as *H_top_* = 32.7 nm.

The material properties used for simulations are provided in Table S1. Unless stated otherwise, the following parameters remained constant in simulations involving the FEM model: side wall thickness 116 nm, *H*_*mat*_ = 170 nm, *H*_*top*_ = 30 nm, *R*_*sub*_ = 5 μm (see Figure 4C for a sketch of the sample geometry).

Figure 5A and B compare 3D FEM simulations and experimental observations when the SU-8 (underlying layer) is excited. The laser is tuned to 1605 cm^–1^ matching the SU-8 absorption making it the heat source. The simulated displacement is shown in color, representing the normalized magnitude, along with the deformed shapes. The cross-sectional profiles from Figures 5A and B show good overlap between the simulation and experimental results, as illustrated in Figure 5C. When the laser is tuned to 1730 cm^–1^, the heat source transitions from the underlying layer to the covering layer. The deformed shapes align well with experimental data (Figures 5D and E), showing strong agreement in the cross-sectional profiles (Figure 5F).

**Figure 5:**
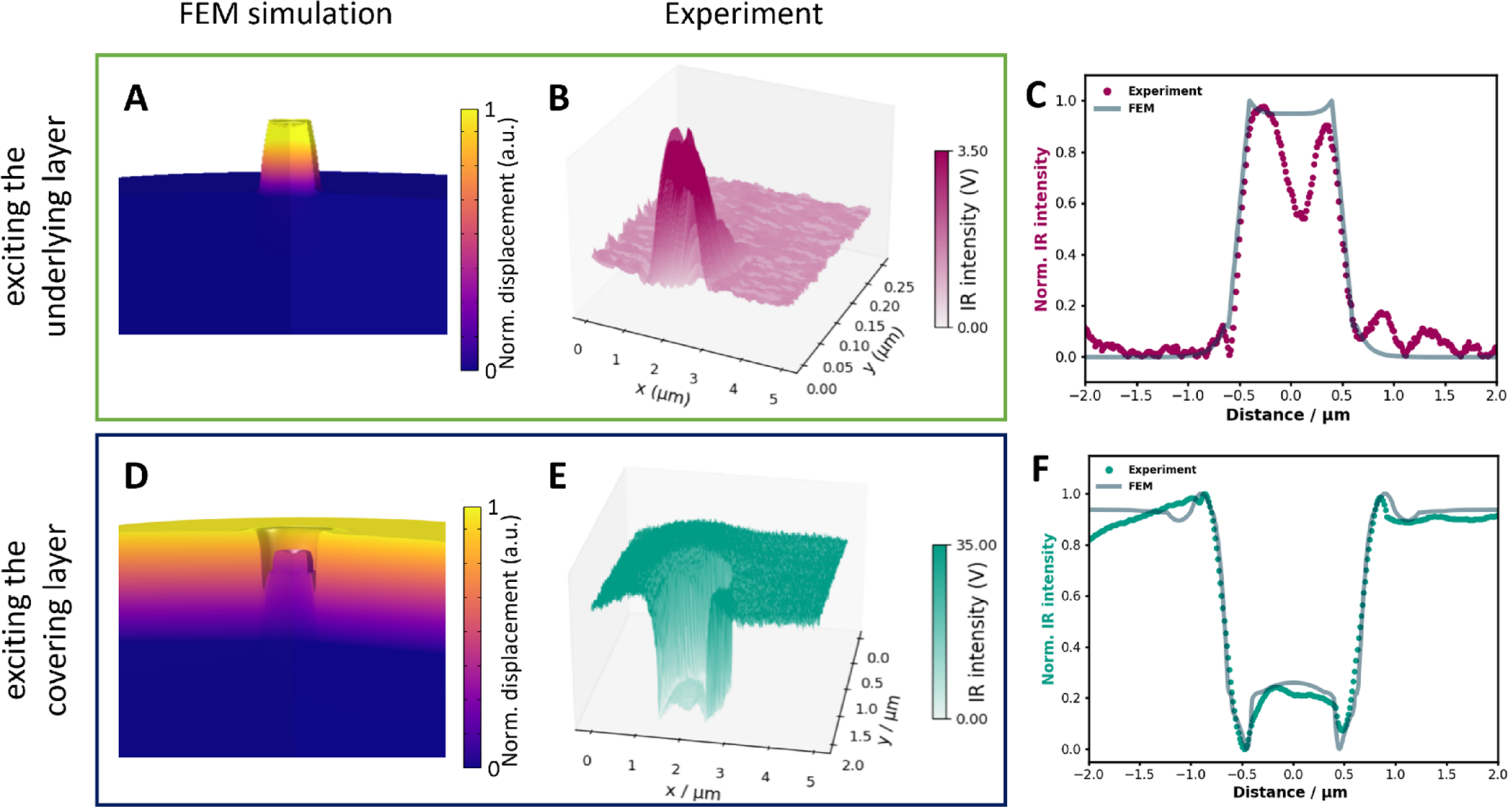
We examine the case of 800 nm pillar. (A) and (D) show 3D FEM simulations of displacement with SU-8 and PMMA as heat sources, respectively, with amplitudes exaggerated for clarity. (B) and (E) represent the corresponding experiments with SU-8 and PMMA excited, respectively. The cross-sectional profiles of the normalized IR intensity from experimental data (B) and (E), compared with the corresponding FEM simulation data from (A) and (D) are shown in (C) and (F), respectively.

Furthermore, by integrating the green areas, as illustrated in Figures S3A and 6A shows that the averaged IR signal at a wavenumber of 1605 cm^–1^ increases nearly linearly with the diameter of SU-8, consistent with FEM simulation results and theoretical prediction (Figure 3A). Likewise, when tuning to 1730 cm^–1^ the signal decreases as the pillar size increases. This means that the lateral extent of the structure affects the local AFM-IR amplitude. In the case of the SU-8 pillar a change by a factor of 2 in size causes an increase of the signal amplitude by 50%.

**Figure 6:**
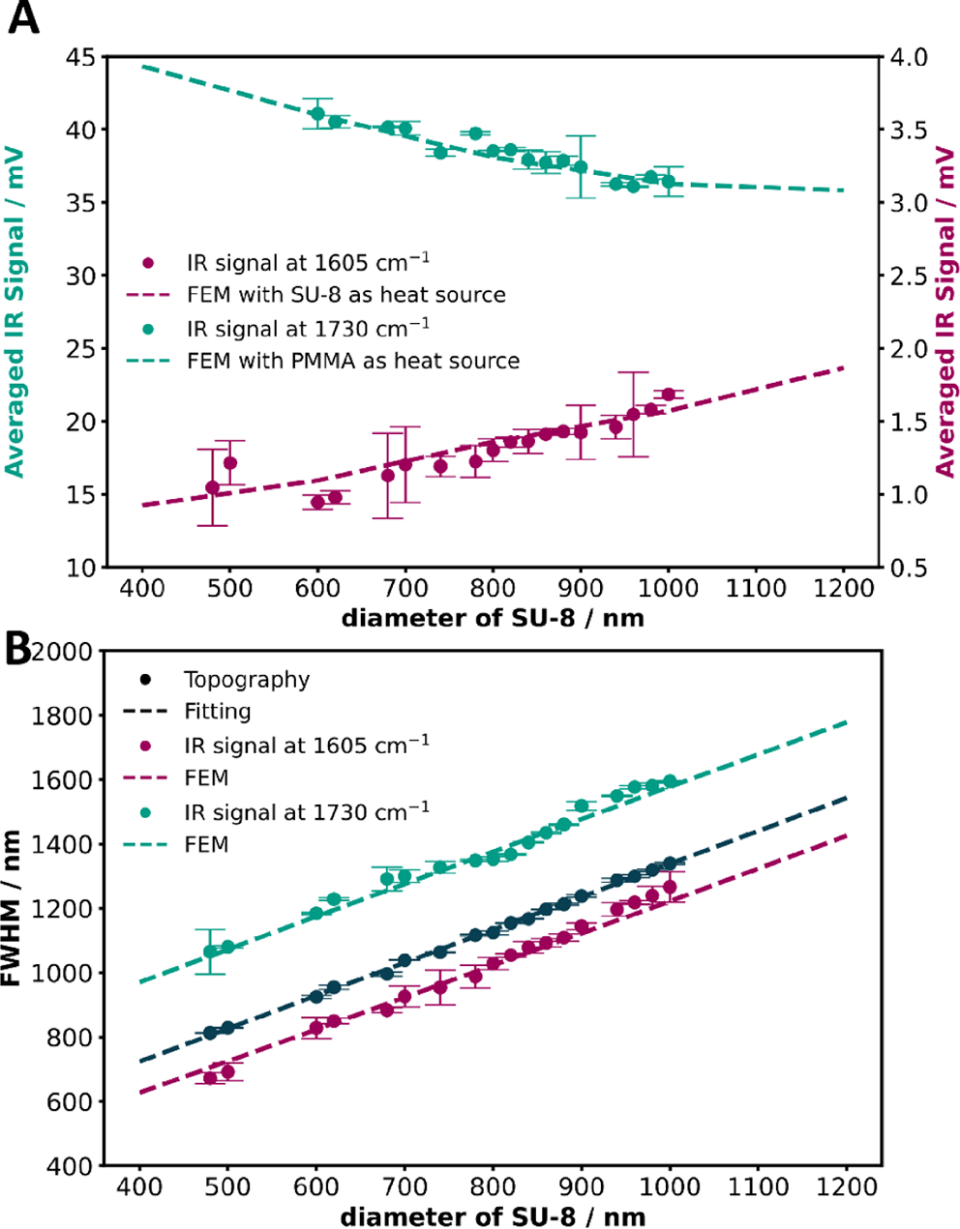
(A) The averaged AFM-IR signal of the examined pillars is plotted as a function of the SU-8 diameter. (B) The fwhm extracted from the cross-sectional profiles of chemical images is shown alongside the corresponding FEM-simulated data. The FEM simulation results were scaled using a consistent arbitrary factor for both SU-8 and PMMA as heat sources, enabling direct comparison with experimental data. Error bars indicate the maximum deviation from the mean values.

The decrease in signal for smaller SU-8 structures may also be influenced by the reduced SU-8/PMMA volume ratio. While the local heating of the SU-8 layer is expected to be similar across different diameters, smaller SU-8 features exert thermal expansion over a relatively larger surrounding PMMA region. This leads to a more distributed strain and consequently a smaller surface deformation, reducing the measured signal.

While the size of the pillar structures in the chemical images increase linearly with feature size, the fwhm of the structure depends on whether the SU-8 subsurface structure or the PMMA covering layer is excited. The same structure when measured at a wavelength absorbed by the subsurface material appears about 358 nm smaller than when measured at a wavelength corresponding to the cover layer, and around 103 nm smaller than the height image. This calculation is based on the intercept difference obtained from the linear fitting of the experimental data. Fitted results are shown in Figure S8. This trend is represented by the experimental data, along with the corresponding simulated lines in Figure 6B, aligning closely with the analytical simulation shown in Figure 3B.

This effect is observed not only in pillar structures but also in more complex geometries, such as the one shown in Figure 7. As before, the structure appears smaller when exciting the subsurface layer (SU-8 at 1035 cm^–1^, see Figure 7A) compared to when exciting the cover layer (PMMA at 1730 cm^–1^, see Figure 7C).

**Figure 7:**
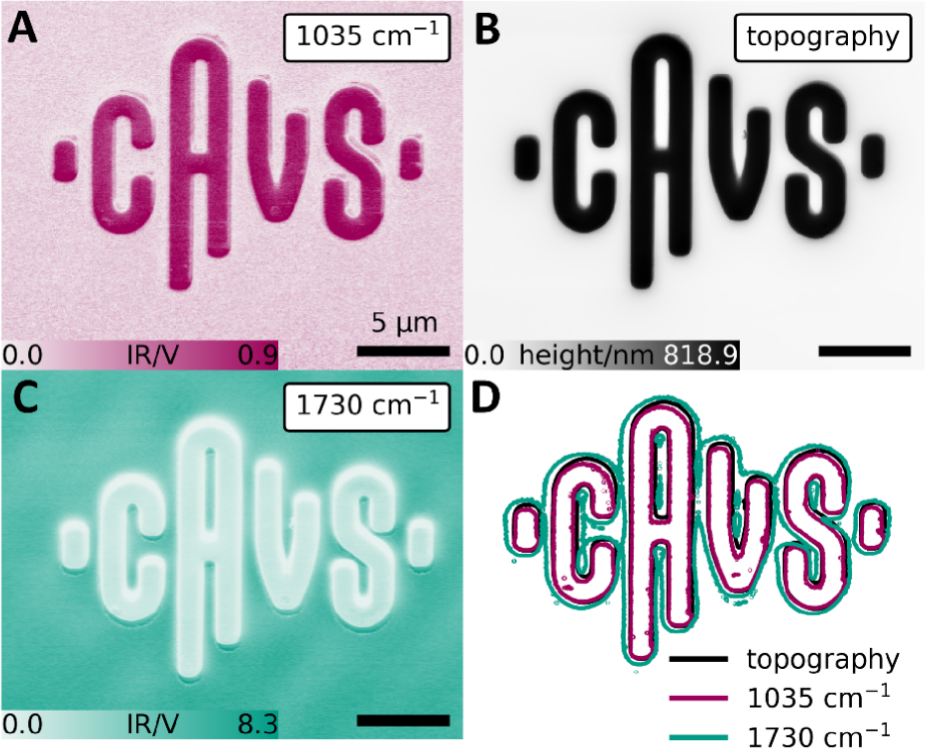
(A) AFM-IR chemical map at 1035 cm^–1^. (B) AFM topography image. (C) Corresponding AFM-IR chemical map at 1730 cm^–1^. (D) Contour lines of the topography image, chemical map at 1035 and 1730 cm^–1^.

Higher IR signals were observed at pillar edges compared to the center, as confirmed by simulations shown in Figure 5C. This phenomenon can be primarily attributed to the tilted, uneven sidewall coverage of the pillar. It is not observed when the SU-8 pillar and the covering layers are vertical (Figure S9A) or when the SU-8 pillar is flush with the surrounding PMMA matrix (Figure S9B). Other contributing factors include differences in thermal expansion coefficients between the materials: PMMA has a thermal expansion coefficient nearly four times higher than that of SU-8. Additionally, the potential presence of interfacial thermal resistance (ITR) between PMMA and SU-8 may also play a role.

To investigate the influence of these factors, we performed FEM simulations, where the SU-8 pillar was modeled as a perfect vertical cylinder, while the PMMA covering layer had a tilted sidewall with the same minimum width as the SU-8 pillar. By varying the tilt angle of the sidewall from 0 to 10 degrees, the results in Figure 8A show that for sidewall angles below 5 degrees, the signal amplitude at the edge of the pillar is significantly higher than at the center. This effect was found to be relatively insensitive to the thermal expansion coefficient. As shown in Figure S7, even when the thermal expansion coefficient of PMMA was assumed to be equal to that of SU-8, the observed signal distribution at the edge and center remained similar.

**Figure 8:**
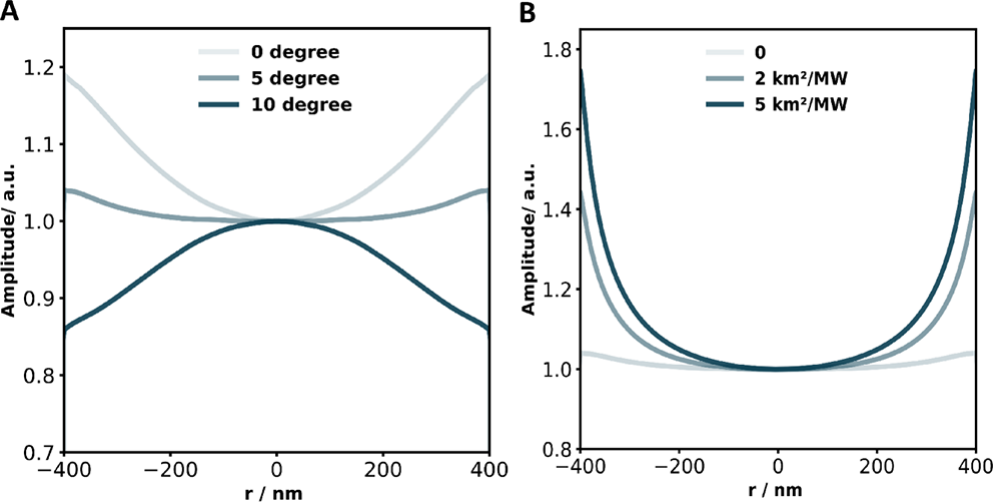
(A) The normalized amplitude of the minimum surface deformation at different radial positions with varying tilted angles of the sidewalls. (B) The normalized amplitude of the minimum surface deformation at different radial positions with varying ITR values.

Additionally, we explored the effect of varying the ITR between the materials. With a 5-degree tilted sidewall, increasing the ITR, resulted in significantly higher signal amplitude at the edge compared to the center, as shown in Figure 8B. While ITR between PMMA and SU-8 may contribute to this effect, it was not explicitly included in our model in order to avoid unnecessary complexity. Future studies quantifying ITR could improve simulation accuracy and provide a more comprehensive understanding of the underlying mechanisms.

## 3 Conclusions and Outlook

In summary, we developed an analytical model to provide a straightforward overview of how sample geometry and material properties influence the AFM-IR signal. While earlier models such as that by Dazzi^32^ focused on localized thermoelastic expansion in homogeneous materials under contact-mode operation, our model extends this framework to multilayer systems, incorporates complex sample geometries, accounts for time-dependent heat diffusion, and includes spatially resolved excitation-features relevant for both contact-mode and tapping-mode AFM-IR. These findings were further verified through experiments and FEM modeling on precisely controlled, fabricated samples.

Our study constitutes the most detailed view of the capability of AFM-IR for subsurface structural analysis yet. We provided experimental evidence supported by theoretical descriptions and modeling of the influence of the surface topography on the chemical image of a subsurface structure for the first time.

A key finding is the variation in the observed structure size in AFM-IR images when exciting the subsurface versus the covering layers caused by the sample geometry. This is observed in our experimental data and also described by the FEM model. Beyond this, the FEM model allows us to explain the detailed features of the AFM-IR signal, attributing them to the surface geometry—specifically, the tilted sidewalls of the pillars.

We also present the first experimental evidence demonstrating that AFM-IR spatial resolution is directly linked to the absorber’s diameter, as demonstrated in Figures 6B and S8. Additionally, we observed a linear increase in signal intensity with absorber diameter. These results align well with the theoretical predictions. We believe these findings directly contribute to improved accuracy when determining the actual size of chemically distinct structures using AFM-IR.

Overall, our findings advance the understanding of AFM-IR’s capabilities, particularly in subsurface imaging and the role of absorber geometry on signal intensity and spatial resolution. These insights pave the way for future research and applications in nanotechnology and materials science.

## 4 Methods

### 4.1 Sample Preparation

After the initial design of the structures, the fabrication process began with spin-coating a SU-8 (SU-8–2) resist layer (approximately 1000 nm thick) onto a silicon wafer and soft-baking at 90 ^◦^C for 300 s. To improve polymer adhesion, the wafer was first cleaned using oxygen plasma treatment, followed by a dehydration baking step at 90 ^◦^C for 300 s. The desired device layout was patterned onto the SU-8 resist using electron beam lithography (EBL) at 100 kV and 50 pA. After exposure, the sample was post baked at 90 ^◦^C for 400 s, developed in an EC-solvent for 60 s and subjected to a final bake at 200 ^◦^C for 30 min. Next, a (185 nm-thick) PMMA (PMMA A4) resist layer was spin-coated over the fabricated sample and hard-baked at 150 ^◦^C for 10 min.

### 4.2 AFM-IR Measurements

All AFM-IR measurements were conducted using a Bruker nano-IR 3s system coupled to a MIRcat-QT external cavity quantum cascade laser array (EC-QCL) from Daylight Solutions, with a spectral range from 910 cm^–1^ to 1950 cm^–1^. Spectra were acquired using AFM-IR in tapping-mode with a heterodyne detection scheme.

For pillar measurements, the cantilever was driven at its first resonance frequency (*f*_1_ ≈ 128 kHz), and the AFM-IR signal was demodulated at the second resonance frequency (*f*_2_ ≈ 839 kHz) using a digital lock-in amplifier (MFLI, Zurich Instruments). The laser repetition rate was adjusted to *f*_L_ = *f*_2_ – *f*_1_ ≈ 711 kHz. An overall gold-coated cantilever with a nominal first free resonance frequency of 150 ± 75 kHz and a nominal spring constant between 5 and 20 N m^–1^ (Tap150GB-G from BudgetSensors) was used. The laser pulse width was set to 220 ns. Laser power was adjusted to 14.75% of the original power using metal mesh attenuators resulting in a pulse peak power of up to 15 mW for 1605 cm^–1^ and 24 mW for 1730 cm^–1^. AFM-IR images were obtained over a 10 μm ×10 μm area at a line-scan rate of 0.1 Hz (lateral speed 100 nm s^–1^) and a resolution of 400 pixels per line.

During measurements of the “CAVS” nanostructure, the cantilever was driven at its first resonance frequency (*f_1_* ≈ 242 kHz), and the AFM-IR signal was demodulated at the second resonance frequency (*f_2_* ≈ 242 kHz) using a digital lock-in amplifier (MFLI, Zurich Instruments). The laser repetition rate was set to *f*_L_ = *f*_2_ – *f*_1_ ≈ 1292 kHz. An overall gold-coated cantilever with a nominal first free resonance frequency of 300 ± 100 kHz and a nominal spring constant between 20 and 75 N m^–1^ (Tap300GB-G from BudgetSensors) was used. The laser was set to emit pulses with a pulse width of 160 ns. Laser power was adjusted to 14.75% of the original power using metal mesh attenuators resulting in a pulse peak power of up to 7 mW for 1035 cm^–1^ and 22 mW for 1730 cm^–1^. For AFM-IR images a 25 μm x 20 μm area was scanned with a line rate of 0.1 Hz (lateral speed 100 nm s^–1^) and a resolution of 800 pixels per line.

All spectra were recorded at each location with a spectral resolution of 1 cm^–1^. Dry air generated by an adsorptive dry air generator was used to purge the instrument and all beam paths.

## Supplementary Material



## Notes

Additional Figures S1 to S9, Table S1 and the additional derivations for equations in the main text is available free of charge. Code and raw data to generate all figures are available on Zenodo (DOI:10.5281/zenodo.14812045)^33^ (PDF)
