# Supplementary material for: Experimental and Theoretical Insights into Nanoscale AFM-IR Imaging of Complex Heterogeneous Structures
Source: Anal Chem. 2025 Sep 18;97(38):21141–9. doi: 10.1021/acs.analchem.5c04707 (PMC12489900; doi:10.1021/acs.analchem.5c04707)
Supplement: Supplementary file 1 [file ac5c04707_si_001.pdf]

SUPPLEMENTARY INFORMATION
Experimental and Theoretical Insights into Nanoscale AFM-IR Imaging of Complex Heterogeneous Structures

Yide Zhang, Ufuk Yilmaz , Artem S.Vorobev, Simone Iadanza, Liam O'Faolain, Bernhard Lendl, and Georg Ramer*

E-mail: georg.ramer@tuwien.ac.at

Y. Zhang, U. Yilmaz, B. Lendl, G. Ramer

Institute of Chemical Technologies and Analytics, TU Wien, Getreidemarkt 9 /E164-02-1, Vienna, 1060, Austria

G. Ramer

Christian Doppler Laboratory for Advanced Mid-Infrared Laser Spectroscopy in (Bio-)process Analytics, TU Wien, Getreidemarkt 9 /E164-02-1, Vienna, 1060, Austria

Y. Zhang, A.S.Vorbev, L. O'Faolain

Centre for Advanced Photonics and Process Analysis, Munster Technological University,Rossa Avenue, Bishopstown, Cork, T12P928, Ireland

S.Iadanza

Laboratory of Nano and Quantum Technologies Paul Scherrer-Institut, Villigen, 5232, Switzerland

Ecole Polytechnique Federale de Lausanne, Lausanne, 1015, Switzerland

Table of Contents

S1 Heat equation in cylindrical coordinates **S3**

S2 Thermo-elastic equation **S13**

S3 Material Properties **S17**

S4 Finite element method modeling **S22**

S5 Code and raw data **S26**

References **S26**

S1 Heat equation in cylindrical coordinates

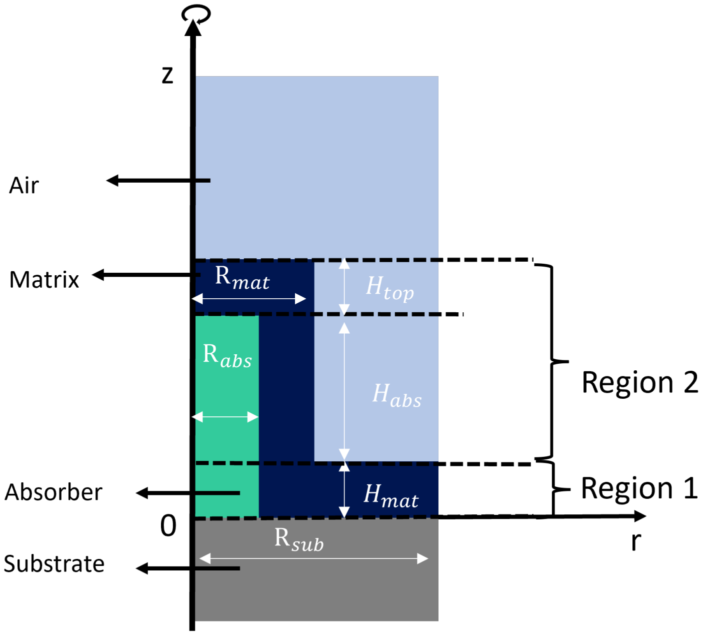

Figure S1: Schematic of the modeled, cylindrically symmetric system composed of a single cylindrical absorber surrounded by a matrix, deposited on a non-absorbing substrate.

We assume that the laser energy is initially absorbed by the absorber based on its absorption coefficient. Consequently, heat is generated internally throughout the solid absorber at a rate of $g(r, z, t)$ per unit volume, which is the product of the optical absorption coefficient and the optical fluence.

The time dependent heating source term can be expressed as

$$g(r, z, t) = g_V(r, z) * \prod \left(\frac{t}{t_p} \right) \tag{S1}$$

where $\prod \left(\frac{t}{t_p} \right)$ is the rectangular box function of length t_p. The sample is coated on a semi-infinite thick substrate, and covered by dry air.

The whole system can be spitted into to parts as shown on Figure S1, region 1 can be treated as either a semi-infinite body over the domain $0 < r < \infty$ or a finite body with a radius of R_{sub}, provided that R_{sub} is sufficiently large enough to contains all possible mode solutions,

with certain thickness H_{mat}. Region 2 can be considered as a limited entity comprising an absorber characterized by its radius R_{abs} and thickness H_{abs}. This absorber is surrounded by a matrix with a radius of R_{mat} and a combined thickness of $H_{abs} + H_{top}$. The whole system is described by Fourier's law with the following initial conditions (IC) and boundary conditions (BC). Footnotes 1 and 2 consistently refer to Region 1 and Region 2, respectively, throughout the text. Here, we assume that the temperature remains consistent at the interface between Region 1 and Region 2.

$$\frac{\partial^2 T(r,z,t)}{\partial r^2} + \frac{1}{r}\frac{\partial T(r,z,t)}{\partial r} + \frac{\partial^2 T(r,z,t)}{\partial z^2} + \frac{g(r,z,t)}{\kappa} = \frac{1}{\alpha}\frac{\partial T(r,z,t)}{\partial t} \quad in \quad t \geq 0 \tag{S2}$$

Region 1 with boundary conditions

$$\begin{aligned} &BC1: \quad \frac{\partial T_1(r,z,t)}{\partial r} = 0 \quad at \quad r = 0 && \text{(symmetry)} \\ &BC2: \quad T_1(r,z,t) = 0 \quad at \quad r = R_{sub} && \text{(constant temperature)} \\ &BC3: \quad T_1(r, z = 0, t) = 0 && \text{(substrate is perfect heat sink)} \\ &IC1: \quad T_1(r,z,t=0) = F_1(r,z) \end{aligned} \tag{S3}$$

Region 2 with boundary conditions

$$\begin{aligned} &BC4: \quad \frac{\partial T_2(r,z,t)}{\partial r} = 0 \quad at \quad r = 0 && \text{(symmetry)} \\ &BC5: \quad \frac{\partial T_2(r,z)}{\partial z} = 0 \quad at \quad z = H_{top} + H_{mat} + H_{abs} && \text{(air is insulating)} \\ &BC6: \quad T_2(z = H_{mat}) = T_1(z = H_{mat}) \\ &IC2: \quad T_2(r,z,t=0) = F_2(r,z) \end{aligned} \tag{S4}$$

t is time, r is the radial distance, κ is the thermal conductivity. In the following, we take room temperature as zero, T is variant above room temperature. α is the thermal diffusivity defined as:

$$\alpha = \frac{\kappa}{\rho C_p} \tag{S5}$$

where ρ is the density and C_p is the specific heat capacity. Note: BC1 is the symmetry condition, BC2 implies that the sample is a finite cylinder with a constant temperature at the sides. This BC2 was chosen over the alternative option of a semi-infinite sample because it allows for a solution that is easier to handle analytically. If R_{sub} is large enough the solutions are almost identical. BC3 and BC5 indicate that the sample is finite in z direction with a constant temperature at sample/substrate interface and insulation condition at top sample/air interface.

To determine the desired Green's function at each region, we consider the homogeneous version of the problem defined above, which we will denote as $\Psi_1(r, z, t)$, for the region 1:

$$\frac{\partial^2 \Psi_1(r,z,t)}{\partial r^2} + \frac{1}{r}\frac{\partial \Psi_1(r,z,t)}{\partial r} + \frac{\partial^2 \Psi_1(r,z,t)}{\partial z^2} = \frac{1}{\alpha}\frac{\partial \Psi_1(r,z,t)}{\partial t} \quad in \quad 0 \le r \le R_{sub}, 0 \le z \le H_{mat} \tag{S6}$$

Now, with all homogeneous boundary conditions, we split our solution into three independent functions

$$\Psi_1(r, z, t) = R_1(r) Z_1(z) \Gamma_1(t) \tag{S7}$$

which after substitution into (S6) yields,

$$\frac{1}{R_1}\left(\frac{d^2 R_1}{dr^2} + \frac{1}{r}\frac{dR_1}{dr}\right) + \frac{1}{Z_1}\frac{d^2 Z_1}{dz^2} = \frac{1}{\alpha \Gamma_1}\frac{d\Gamma}{dt} = -\lambda_1^2 \tag{S8}$$

Solution of separated ODE in the t dimension yields the expected form

$$\Gamma_1(t) = C_1 e^{-\alpha \lambda_1^2 t} \tag{S9}$$

with the remaining terms of (S8) yielding

$$\frac{1}{R_1}\left(\frac{d^2 R_1}{dr^2} + \frac{1}{r}\frac{dR_1}{dr}\right) + \lambda_1^2 = -\frac{1}{Z_1}\frac{d^2 Z_1}{dz^2} = \eta_1^2 \tag{S10}$$

Solution of the z-dimension ODE yields the desired solution form

$$Z_1(z) = C_2 \cos \eta z + C_3 \sin \eta z \tag{S11}$$

Applying BC3 yields constant $C_2 = 0$. We now consider the remaining r terms of (S10), where we first let $\beta^2 = \lambda^2 - \eta^2$, and then multiply both sides by function R, yielding

$$\frac{d^2 R_1}{dr^2} + \frac{1}{r}\frac{dR_1}{dr} + \beta^2 R_1 = 0 \tag{S12}$$

This is the Bessel equation of order zero, and the elementary solutions are[S1]

$$R_1(r) = C_4 J_0(\beta r) + C_5 Y_0(\beta r) \tag{S13}$$

where $J_0(\beta r)$ and $Y_0(\beta r)$ are Bessel functions of order zero. The requirement of symmetry condition stated by BC1 eliminates the $Y_0(\beta_1 r)$ term, while BC2 then yields

$$C_4 J_0(\beta_m R_{sub}) = 0 \quad for \quad m = 0, 1, 2, 3, .. \tag{S14}$$

Now λ_m is,

$$\lambda_m^2 = \beta_m^2 + \eta^2 \tag{S15}$$

having defined the eigenvalues and eigenfunctions for both spatial dimensions, we form a product solution of the separated functions and sum over all possible solutions, yielding

$$\Psi_1(r, z, t) = \sum_{m=0}^{\infty} C_m J_0(\beta_m r) sin(\eta z) e^{-\alpha \lambda_m^2 t} \tag{S16}$$

The initial condition applied yielding

$$\Psi_1(t = 0) = F_1(r, z) = \sum_{m=0}^{\infty} C_m J_0(\beta_m r) sin(\eta z) \tag{S17}$$

For region 2, we use the same methods for region 1 described above, which we will denote as $\Psi_2(r,z,t)$, for the region 2:

$$\frac{\partial^2\Psi_2(r,z,t)}{\partial r^2}+\frac{1}{r}\frac{\partial\Psi_2(r,z,t)}{\partial r}+\frac{\partial^2\Psi_2(r,z,t)}{\partial z^2}=\frac{1}{\alpha}\frac{\partial\Psi_2(r,z,t)}{\partial t}$$
$$in \quad 0\le r\le R_{mat}, H_{mat}\le z\le H_{top}+H_{mat}+H_{abs} \tag{S18}$$

Now, with all homogeneous boundary conditions, we split our solution into three independent functions

$$\Psi_2(r,z,t)=R_2(r)Z_2(z)\Gamma_2(t) \tag{S19}$$

Applying BC6, we consider the solution of $Z_2(z)$ contains the same function of $Z_1(z)$, therefore,

$$Z_2(z)=C_3\sin\eta z \tag{S20}$$

while applying BC6 yields the eigenvalues for integer values of n

$$\eta=\frac{\pi}{2(H_{abs}+H_{mat}+H_{top})}(2n+1),\quad n=0,1,2,... \tag{S21}$$

The requirement of symmetry condition stated by BC4 eliminates the $Y_0(\gamma r)$ term of the solution $R_2(r)$. Meanwhile, in order to satisfy BC5, we assume $R_1(r)=R_2(r)$ in the area $0\le r\le R_{mat}$, then yields the same eigenvalues in radial direction

$$\gamma_l=\beta_m \tag{S22}$$

Since region 1 and region 2 share same eigenvalues, we replace γ_l by β_m, λ_{mn} by λ_{mn} in the following. Now λ_{mn} is,

$$\lambda_{mn}^2=\beta_m^2+\eta_n^2, \tag{S23}$$

having defined the eigenvalues and eigenfunctions for both spatial dimensions, we form a

product solution of the separated functions and sum over all possible solutions, yielding

$$\Psi_2(r,z,t) = \sum_{n=0}^{\infty}\sum_{m=0}^{\infty} C_{mn} J_0(\beta_m r) sin(\eta_n z) e^{-\alpha\lambda_{mn}^2 t} \tag{S24}$$

For region 1, to find the Fourier coefficients, we use the property of orthogonality of our two enigenfunctions over their respective intervals by applying successively the following operators to both sides of Eq. S17[S2]:

$$* \int_{z=0}^{H_{mat}} sin(\eta_i z) dz \quad and * \int_{r=0}^{R_{sub}} r J_0(\beta_j r) dr \tag{S25}$$

which yields,

$$C_{mn} = \frac{\int_{z=0}^{H_{mat}} \int_{r=0}^{R_{sub}} F_1(r,z) r J_0(\beta_m r) sin(\eta_n z) dr dz}{\int_{z=0}^{H_{mat}} \int_{r=0}^{R_{sub}} r J_0^2(\beta_m r) sin^2(\eta_n z) dr dz} \tag{S26}$$

The solution of the integrals in the denominator are[S3]

$$\int_{r=0}^{R_{sub}} r J_0(\beta_m r) dr = \frac{R_{sub}}{\beta_m} J_1(\beta_m R_{sub}) \tag{S27}$$

$$\int_{r=0}^{R_{sub}} r J_0^2(\beta_m r) dr = \frac{R_{sub}^2}{2} J_1^2(\beta_m R_{sub}) \tag{S28}$$

$$\int_{z=0}^{H_{mat}} sin^2(\eta_n z) dz = \frac{H_{mat}}{2} - \frac{sin(2\eta_n H_{mat})}{4\eta_n} = \frac{2\eta_n H_{mat} - sin(2\eta_n H_{mat})}{4\eta_n} \tag{S29}$$

The solution in our general form, introducing the Fourier constants, namely,

$$\begin{aligned} \Psi_1(r,z,t) = \sum_{n=0}^{\infty}\sum_{m=0}^{\infty} & \frac{8\eta_n J_0(\beta_m r) sin(\eta_n z) e^{-\alpha\lambda_{mn}^2 t}}{(2\eta_n H_{mat} - sin(2\eta_n H_{mat})) R_{sub}^2 J_1^2(\beta_m R_{sub})} \\ & \times \int_{z'=0}^{H_{mat}} \int_{r'=0}^{R_{sub}} F(r',z') r' J_0(\beta_m r') sin(\eta_n z') dr' dz' \end{aligned} \tag{S30}$$

We now reformulate the above solution into

$$\Psi_1(r,z,t) = \int_{z'=0}^{H_{mat}} \int_{r'=0}^{R_{sub}} \left[\sum_{n=0}^{\infty} \sum_{m=0}^{\infty} \frac{8\eta_n J_0(\beta_m r) sin(\eta_n z) e^{-\alpha \lambda_{mn}^2 t}}{(2\eta_n H_{mat} - sin(2\eta_n H_{mat})) R_{sub}^2 J_1^2(\beta_m R_{sub})} J_0(\beta_m r') sin(\eta_n z') \right] \times F(r',z') r' dr' dz' \tag{S31}$$

The solution of the homogeneous problem (S6) in terms of Green's function is given as

$$\Psi_1(r,z,t) = \int_{z'=0}^{H_{mat}} \int_{r'=0}^{R_{sub}} G_1(r,z,t|r',z',t')|_{t'=0} F_1(r',z') r' dr' dz' \tag{S32}$$

By comparing this solution with (S30), we readily conclude that $G(r,z,t|r',z',t')|_{t'=0}$ is given by

$$G_1(r,z,t|r',z',t')|_{t'=0} = \sum_{n=0}^{\infty} \sum_{m=0}^{\infty} \frac{8\eta_n J_0(\beta_m r) sin(\eta_n z) e^{-\alpha \lambda_{mn}^2 t}}{(2\eta_n H_{mat} - sin(2\eta_n H_{mat})) R_{sub}^2 J_1^2(\beta_m R_{sub})} J_0(\beta_m r') sin(\eta_n z') e^{-\alpha \lambda_{mn}^2 t} \tag{S33}$$

Green's function $G(r,z,t|r',z',t')$ is now determined by replacing t with $t-t'$, yielding the result[S2]

$$G_1(r,z,t|r',z',t') = \sum_{n=0}^{\infty} \sum_{n=0}^{\infty} \sum_{m=0}^{\infty} \frac{8\eta_n J_0(\beta_m r) sin(\eta_n z) e^{-\alpha \lambda_{mn}^2 t}}{(2\eta_n H_{mat} - sin(2\eta_n H_{mat})) R_{sub}^2 J_1^2(\beta_m R_{sub})} J_0(\beta_m r') sin(\eta_n z') e^{-\alpha \lambda_{mn}^2 (t-t')} \tag{S34}$$

Then the solution of the non-homogeneous problem of (S2) in terms of the above Greens's function is given, according to (S34) as[S2]

$$\begin{aligned} T_1(r,z,t) = &\int_{z'=0}^{H_{mat}} \int_{r'=0}^{R_{sub}} G_1(r,z,t|r',z',t')|_{t'=0} F_1(r',z') r' dr' dz' \\ &+ \frac{\alpha}{\kappa} \int_{t'>0}^{t} \int_{z'=0}^{H_{mat}} \int_{r'=0}^{R_{sub}} G(r,z,t|r',z',t') g_1(r',z',t') r' dr' dz' \end{aligned} \tag{S35}$$

In our case, the medium is initially at room temperature with zero temperature variation, and temperature at boundary is not maintained. So $F(r,z) = 0$ then the solution of the

non-homogeneous problem defined by (S2) is given in terms of the Green's function becomes in variable form:

$$T_1(r,z,t) = \frac{\alpha}{\kappa}\int_{t'>0}^{t}\int_{z'=0}^{H_{mat}}\int_{r'=0}^{R_{sub}} G(r,z,t|r',z',t')g_1(r',z',t')r'dr'dz' \tag{S36}$$

Introducing the Green's function of (S34) into (S36). We define the heat source as

$$g_1(r',z',t') = g_V(r',z') * \prod(\frac{t'}{t_p}), \quad in \quad 0 \leq r' \leq R_{abs}, 0 \leq z' \leq H_{mat}, t \geq 0 \tag{S37}$$

Substitution of the above equation and Green's function (S34) into (S36) yielding the overall temperature solution

$$\begin{aligned} T_1(r,z,t) =& \frac{\alpha}{\kappa}\int_{t'=0}^{t_p}\int_{z'=0}^{H_{mat}}\int_{r'=0}^{R_{abs}}\sum_{n=0}^{\infty}\sum_{m=0}^{\infty}\frac{8\eta_n J_0(\beta_m r)sin(\eta_n z)e^{-\alpha\lambda_{mn}^2 t}}{(2\eta_n H_{mat} - sin(2\eta_n H_{mat}))R_{sub}^2 J_1^2(\beta_m R_{sub})} \\ & \times r'J_0(\beta_m r')sin(\eta_n z')e^{-\alpha\lambda_{mn}^2(t-t')}g_V(r',z')\prod(\frac{t'}{t_p})dr'dz'dt' \\ =& \sum_{n=0}^{\infty}\sum_{m=0}^{\infty}A_1(\beta_m,\eta_n)J_0(\beta_m r)sin(\eta_n z)\begin{cases} 1 - e^{-\alpha\lambda_{mn}^2 t} & \text{in} \quad 0 < t \leq t_p \\ \\ (e^{\alpha\lambda_{mn}^2 t_p} - 1)e^{-\alpha\lambda_{mn}^2 t} & \text{for} \quad t > t_p \end{cases} \end{aligned} \tag{S38}$$

where $A_1(\beta_m,\eta_n) = \frac{8g_V R_{abs}}{\kappa(2\eta_n H_{mat} - sin(2\eta_n H_{mat}))R_{sub}^2}\frac{J_1(\beta_m R_{abs})(1-cos(\eta_n H_{mat}))}{J_1^2(\beta_m R_{sub})\lambda_{mn}^2\beta_m}$.

For region 2, the initial condition applied yielding

$$\Psi_2(t=0) = F_2(r,z) = \sum_{m=0}^{\infty}\sum_{n=0}^{\infty}C_{mn}J_0(\beta_m r)sin(\eta_n z) \tag{S39}$$

To find the Fourier coefficients, we use the property of orthogonality of our two enigenfunctions over their respective intervals by applying successively the following operators to both sides of Eq. S39:

$$*\int_{z=H_{mat}}^{H_{abs}+H_{mat}+H_{top}} sin(\eta_i z)dz \quad and * \int_{r=0}^{R_{mat}} rJ_0(\gamma_j r)dr \tag{S40}$$

which yields,

$$C_{mn} = \frac{\int_{z=H_{mat}}^{H_{abs}+H_{mat}+H_{top}} \int_{r=0}^{R_{mat}} F_2(r,z) r J_0(\gamma_m r) sin(\eta_n z) dr dz}{\int_{z=H_{mat}}^{H_{abs}+H_{mat}+H_{top}} \int_{r=0}^{R_{mat}} r J_0^2(\beta_m r) sin^2(\eta_n z) dr dz} \tag{S41}$$

The solution of the integrals in the denominator are[S3]

$$\int_{r=0}^{R_{mat}} r J_0(\beta_m r) dr = \frac{R_{mat}}{\beta_m} J_1(\beta_m R_{mat}) \tag{S42}$$

$$\int_{r=0}^{R_{mat}} r J_0^2(\beta_m r) dr = \frac{R_{mat}^2}{2} J_1^2(\beta_m R_{mat}) \tag{S43}$$

$$\int_{z=H_{mat}}^{H_{abs}+H_{mat}+H_{top}} sin^2(\eta_n z) dz = \frac{H_{abs}+H_{top}}{2} + \frac{sin(2\eta_n H_{mat})}{4\eta_n} = \frac{2\eta_n(H_{top}+H_{abs}) + sin(2\eta_n H_{mat})}{4\eta_n} \tag{S44}$$

The solution in our general form, introducing the Fourier constants, namely,

$$\begin{aligned}\Psi_2(r,z,t) = &\sum_{n=0}^{\infty}\sum_{m=0}^{\infty} \frac{8\eta_n J_0(\beta_m r) sin(\eta_n z) e^{-\alpha\lambda_{mn}^2 t}}{(2\eta_n(H_{abs}+H_{top}) + sin(2\eta_n H_{mat})) R_{mat}^2 J_1^2(\beta_m(R_{mat}))} \\ &\times \int_{z'=H_{mat}}^{H_{abs}+H_{mat}+H_{top}} \int_{r'=0}^{R_{mat}} F_2(r',z') r' J_0(\beta_m r') sin(\eta_n z') dr' dz'\end{aligned} \tag{S45}$$

We now reformulate the above solution into

$$\begin{aligned}\Psi_2(r,z,t) = &\int_{z'=H_{mat}}^{H_{abs}+H_{mat}+H_{top}} \int_{r'=0}^{R_{mat}} \left[\sum_{n=0}^{\infty}\sum_{m=0}^{\infty} \frac{8\eta_n J_0(\beta_m r) sin(\eta_n z) J_0(\beta_m r') sin(\eta_n z') e^{-\alpha\lambda_{mn}^2 t}}{(2\eta_n(H_{abs}+H_{top}) + sin(2\eta_n H_{mat})) R_{mat}^2 J_1^2(\beta_m R_{mat})}\right] \\ &\times F_2(r',z') r' dr' dz'\end{aligned} \tag{S46}$$

The solution of the homogeneous problem (S18) in terms of Green's function is given as

$$\Psi_2(r,z,t) = \int_{z'=0}^{L} \int_{r'=0}^{b} G_2(r,z,t|r',z',t')|_{t'=0} F_2(r',z') r' dr' dz' \tag{S47}$$

By comparing this solution with (S45), we readily conclude that $G_2(r,z,t|r',z',t')|_{t'=0}$ is

given by

$$G_2(r,z,t|r',z',t')|_{t'=0} = \sum_{n=0}^{\infty}\sum_{m=0}^{\infty} \frac{8\eta_n J_0(\beta_m r) sin(\eta_n z) J_0(\beta_m r') sin(\eta_n z') e^{-\alpha\lambda_{mn}^2 t}}{(2\eta_n(H_{abs}+H_{top}) + sin(2\eta_n H_{mat}))R_{mat}^2 J_1^2(\beta_m R_{mat})} \quad \text{(S48)}$$

Green's function $G_2(r,z,t|r',z',t')$ is now determined by replacing t with $t-t'$, yielding the result[S2]

$$G_2(r,z,t|r',z',t') = \sum_{n=0}^{\infty}\sum_{m=0}^{\infty} \frac{8\eta_n J_0(\beta_m r) sin(\eta_n z) J_0(\beta_m r') sin(\eta_n z')}{(2\eta_n(H_{abs}+H_{top}) + sin(2\eta_n H_{mat}))R_{mat}^2 J_1^2(\beta_m R_{mat})} e^{-\alpha\lambda_{mn}^2 (t-t')} \quad \text{(S49)}$$

Then the solution of the non-homogeneous problem of (S2) in terms of the above Greens's function is given, according to (S34) as

$$\begin{aligned} T_2(r,z,t) =& \frac{\alpha}{\kappa}\int_{t'=0}^{t_p}\int_{z'=H_{mat}}^{H_{abs}+H_{mat}}\int_{r'=0}^{R_{abs}} \sum_{n=0}^{\infty}\sum_{m=0}^{\infty} \frac{8\eta_n J_0(\beta_m r) sin(\eta_n z)}{(2\eta_n(H_{abs}+H_{top}) + sin(2\eta_n H_{mat}))R_{mat}^2 J_1^2(\beta_m R_{mat})} \\ &\times r' J_0(\beta_m r') sin(\eta_n z') e^{-\alpha\lambda_{mn}^2(t-t')} g_V(r',z') \prod(\frac{t'}{t_p}) dr' dz' dt' \\ =& \sum_{n=0}^{\infty}\sum_{m=0}^{\infty} A_2(\beta_m,\eta_n) J_0(\beta_m r) sin(\eta_n z) \begin{cases} 1 - e^{-\alpha\lambda_{mn}^2 t} & \text{in} \quad 0 \le t \le t_p \\ (e^{\alpha\lambda_{mn}^2 t_p} - 1) e^{-\alpha\lambda_{mn}^2 t} & \text{for} \quad t > t_p \end{cases} \end{aligned} \quad \text{(S50)}$$

where $A_2(\beta_m,\eta_n) = \frac{8 g_V R_{abs}}{\kappa(2\eta_n(H_{abs}+H_{top}) + sin(2\eta_n H_{mat}))R_{mat}^2} \frac{J_1(\beta_m R_{abs})(cos(\eta_n H_{mat}) - cos(\eta_n(H_{abs}+H_{mat}))}{J_1^2(\beta_m R_{mat})\lambda_{mn}^2 \beta_m}$. The total temperature change is the summation of $T_1(r,z,t)$ and $T_2(r,z,t)$,

$$\begin{aligned} T(r,z,t) &= T_1(r,z,t) + T_2(r,z,t) \\ &= \sum_{n=0}^{\infty}\sum_{m=0}^{\infty} A(\beta_m,\eta_n) J_0(\beta_m r) sin(\eta_n z) \mathcal{T}(t) \end{aligned} \quad \text{(S51)}$$

where

$$
\begin{aligned}
&A(\beta_m,\eta_n) = A_1(\beta_m,\eta_n) + A_2(\beta_m,\eta_n)\\
&= \frac{8g_V R_{abs} J_1(\beta_m R_{abs})}{\kappa \lambda_{mn}^2 \beta_m}\\
&\times \left(\frac{1-cos(\eta_n H_{mat})}{(2\eta_n H_{mat} - sin(2\eta_n H_{mat}))R_{sub}^2 J_1^2(\beta_m R_{sub})} - \frac{cos(\eta_n H_{mat}) - cos(\eta_n(H_{abs}+H_{mat})}{(2\eta_n(H_{abs}+H_{top}) + sin(2\eta_n H_{mat}))R_{mat}^2 J_1^2(\beta_m R_{mat})} \right)
\end{aligned}
\tag{S52}
$$

and time dependent term is

$$
\mathcal{T}(t) = \begin{cases} 1 - e^{-\alpha\lambda_{mn}^2 t} & \text{in} \quad 0 \le t \le t_p \\ \\ (e^{\alpha\lambda_{mn}^2 t_p} - 1)e^{-\alpha\lambda_{mn}^2 t} & \text{for} \quad t > t_p \end{cases} \tag{S53}
$$

S2 Thermo-elastic equation

Following Noda et al,[S4] the Navier's equations for axisymmetric thermoelastic problems without the body forces can be expressed as

$$
\bigtriangledown^2 u_z + \frac{1}{1-2v}\frac{\partial e}{\partial z} - 2\alpha_z(\frac{1+v}{1-2v})\frac{\partial T}{\partial z} = 0 \tag{S54}
$$

where

$$
\bigtriangledown^2 = \frac{\partial^2}{\partial r^2} + \frac{1}{r}\frac{\partial}{\partial r} + \frac{\partial^2}{\partial z^2} \tag{S55}
$$

where E Young's modulus, α_z coefficient of linear thermal expansion, v Poisson ratio. e is the dilatation defined by the sum of strain components

$$
e = \epsilon_{xx} + \epsilon_{yy} + \epsilon_{zz} \tag{S56}
$$

The solution of Naviers equations without body forces can be expressed by Goodiers

thermoelastic displacement potential Φ and Boussinesq harmonic functions φ and ψ under the axisymmetric conditions.

$$u_z = \frac{\partial \Phi}{\partial z} + \frac{\partial \varphi}{\partial z} + z\frac{\partial \psi}{\partial z} - (3-4v)\psi \tag{S57}$$

in which the Goodiers thermoelastic displacement potential Φ must satisfy the governing equations

$$\bigtriangledown^2 \Phi = KT, \tag{S58}$$

where K is Restraint coefficient defined as[S4]

$$K = \frac{\beta}{\lambda + 2\mu} = \left(\frac{1+v}{1-v}\right)\alpha_z \tag{S59}$$

where β is the thermoelastic constant, λ and μ are the Lamé elastic constants, α_z coefficient of the linear thermal expansion. Boussinesq harmonic functions φ and ψ must satisfy the governing equations

$$\bigtriangledown^2 \varphi = \frac{\partial^2 \varphi}{\partial r^2} + \frac{1}{r}\frac{\partial \varphi}{\partial r} + \frac{\partial^2 \varphi}{\partial z^2} = 0 \tag{S60}$$

$$\bigtriangledown^2 \psi = \frac{\partial^2 \psi}{\partial r^2} + \frac{1}{r}\frac{\partial \psi}{\partial r} + \frac{\partial^2 \psi}{\partial z^2} = 0 \tag{S61}$$

The components of the stress are represented by the Goodier thermoelastic displacement potential Φ and Boussinesq harmonic functions φ, ψ are:

$$\sigma_{rr} = 2G\left(\frac{\partial^2 \Phi}{\partial r^2} - KT + \frac{\partial^2 \varphi}{\partial r^2} + z\frac{\partial^2 \psi}{\partial r^2} - 2v\frac{\partial \psi}{\partial z}\right) \tag{S62}$$

$$\sigma_{\theta\theta} = 2G\left(\frac{1}{r}\frac{\partial\Phi}{\partial r} - KT + \frac{1}{r}\frac{\partial\varphi}{\partial r} + \frac{z}{r}\frac{\partial\psi}{\partial r} - 2v\frac{\partial\psi}{\partial z}\right) \tag{S63}$$

$$\sigma_{zz} = 2G\left[\frac{\partial^2\Phi}{\partial z^2} - KT + \frac{\partial^2\varphi}{\partial z^2} + z\frac{\partial^2\psi}{\partial z^2} - 2(1-v)\frac{\partial\psi}{\partial z}\right] \tag{S64}$$

$$\sigma_{zr} = 2G\left[\frac{\partial^2\Phi}{\partial r\partial z} + \frac{\partial^2\varphi}{\partial r\partial z} + z\frac{\partial^2\psi}{\partial r\partial z} - (1-2v)\frac{\partial\psi}{\partial r}\right] \tag{S65}$$

Referring to Eq.(S61) and (S60), the functions φ and ψ can be expressed by use of integrals satisfying the solution form as shown on[S4] under the axial symmetric condition,

$$\varphi = \sum_{n=0}^{\infty}\sum_{m=0}^{\infty} BJ_0(\beta_m r)e^{-\beta_m z} \tag{S66}$$

$$\psi = \sum_{n=0}^{\infty}\sum_{m=0}^{\infty} CJ_0(\beta_m r)e^{-\beta_m z} \tag{S67}$$

The Goodier thermoelastic displacement potential Φ can be expressed as,

$$\Phi(r,z,t) = \sum_{n=0}^{\infty}\sum_{m=0}^{\infty} \frac{-A(\beta_m,\eta_n)K}{\beta_m^2+\eta_n^2} J_0(\beta_m r)sin(\eta_n z)\mathcal{T}(t) \tag{S68}$$

Substituting Eq.(S66), (S67) and (S68) into (S57), the displacement is

$$u_z(r,z,t) = \sum_{n=0}^{\infty}\sum_{m=0}^{\infty}\left[\frac{-A(\beta_m,\eta_n)K\eta_n}{\beta_m^2+\eta_n^2}J_0(\beta_m r)cos(\eta_n z)\mathcal{T}(t) + (-(B+zC)\beta_m - (3-4v)C)J_0(\beta_m r)e^{-\beta_m z}\right] \tag{S69}$$

The boundary conditions on the traction free surface are

$$\sigma_{zz} = 0, \quad \sigma_{zr} = 0 \quad on \quad z = H_{mat} \tag{S70}$$

The unknown functions B and C can be determined from the boundary conditions (S70)

$$C = -\frac{A(\beta_m,\eta_n)K}{\beta_m e^{-\beta_m H_{mat}}}\left(\frac{\eta_n^2}{\beta_m^2+\eta_n^2}+1\right)sin(\eta_n z)\mathcal{T}(t) \tag{S71}$$

$$B = -\frac{1-2v+H_{mat}\beta_m}{\beta_m}C \tag{S72}$$

Substituting Eq.(S72) and (S71) into (S69) the displacements can be simplified as,

$$u_z(r,z,t) = \sum_{n=0}^{\infty}\sum_{m=0}^{\infty}\frac{2(1+v)A(\beta_m,\eta_n)\alpha_z}{\beta_m}\left(\frac{\eta_n^2}{\beta_m^2+\eta_n^2}+1\right)J_0(\beta_m r)sin(\eta_n z)\mathcal{T}(t) \tag{S73}$$

at surface,

$$u_z(r,t) = \sum_{n=0}^{\infty}\sum_{m=0}^{\infty}\frac{2(1+v)A(\beta_m,\eta_n)\alpha_z}{\beta_m}\left(\frac{\eta_n^2}{\beta_m^2+\eta_n^2}+1\right)J_0(\beta_m r)\mathcal{T}(t) \tag{S74}$$

S3 Material Properties

Table S1: Parameters used in finite-difference time domain model and analytical model of AFM-IR simulations. The thermal, mechanical and thermo-mechanical properties listed below.

Property*	PMMA	SU-8	air	silicon
absorption coefficient (1/cm)	960[S5]	43[S6]	-	-
coefficient of thermal expansion (1/k)	193.6×10^{-6}[S7]	52×10^{-6}[S8]	-	2.6×10^{-6}[S9]
thermal conductivity (W/(m· k))	0.192[S10]	0.2[S11]	0.025[S12]	130[S9]
density (kg/m^3)	1190[S13]	1219[S14]	1.2[S12]	2329[S9]
heat capacity (J/(kg·k))	1420[S15]	1200[S14]	1015[S12]	700[S9]
Young's modulus (GPa)	2.4[S16]	4.95[S17]	-	170[S18]
Poisson's ratio	0.37[S16]	0.22[S19]	-	0.28[S18]

*These values are treated as exact numbers for the purpose of simulations.

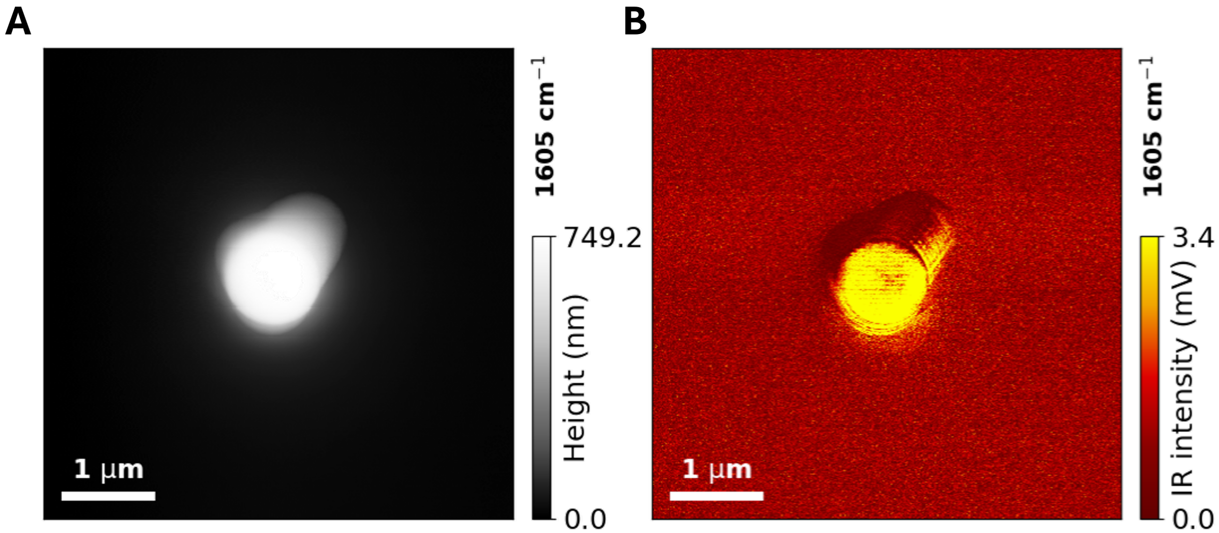

Figure S2: (A) AFM topography image of a single SU-8 nanopillar with a diameter of 800 nm. (B) Corresponding AFM-IR chemical map at 1605 cm^{-1}.

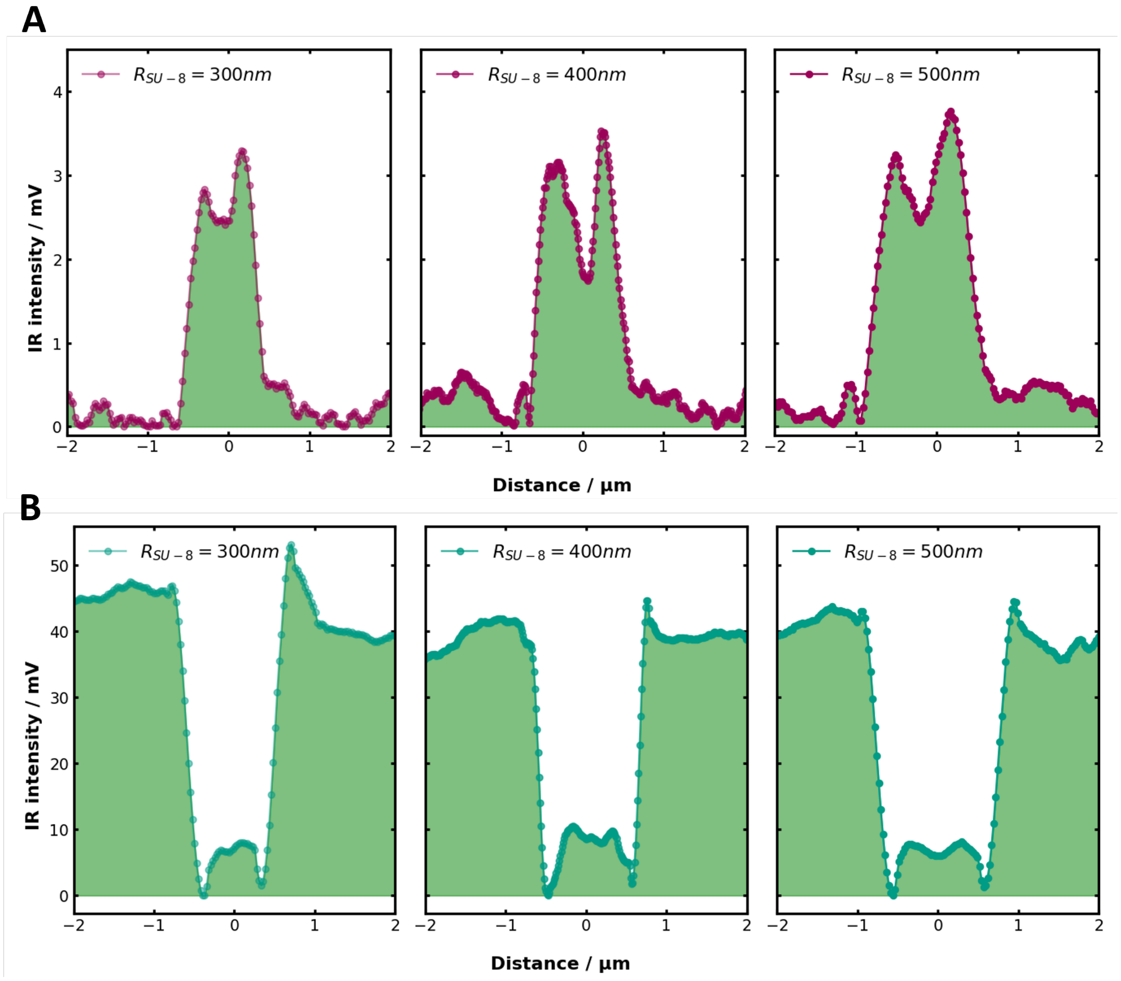

Figure S3: (A) Cross-sectional profiles at different radii of SU-8 were obtained from the AFM-IR chemical map at 1605 cm^{-1}. (B) Cross-sectional profiles at different radii of SU-8 were obtained from the AFM-IR chemical map at 1730 cm^{-1}.

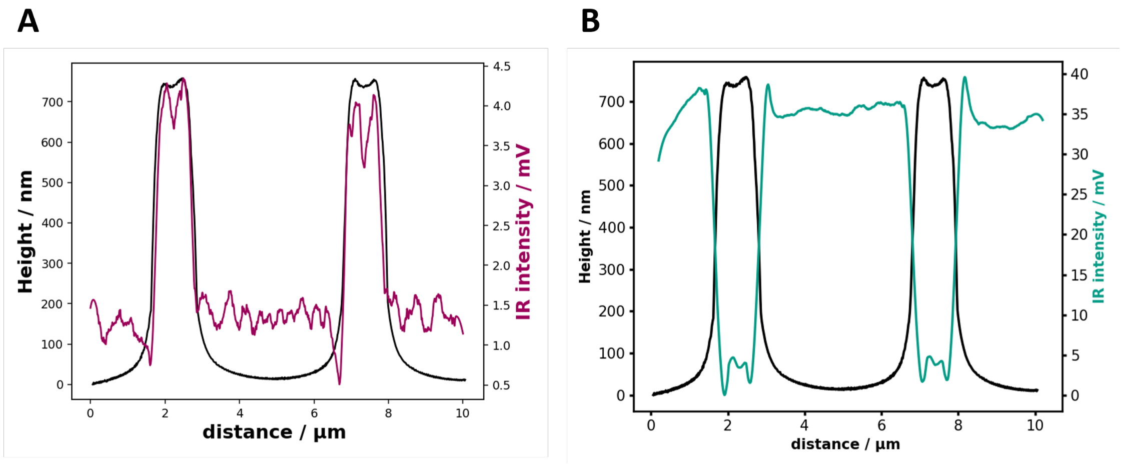

Figure S4: (A) Cross-sectional profile of the initial row in the topography image and the chemical image at 1605 cm^{-1}. (B) Cross-sectional profile of the initial row in the topography image and the chemical image at 1730 cm^{-1}.

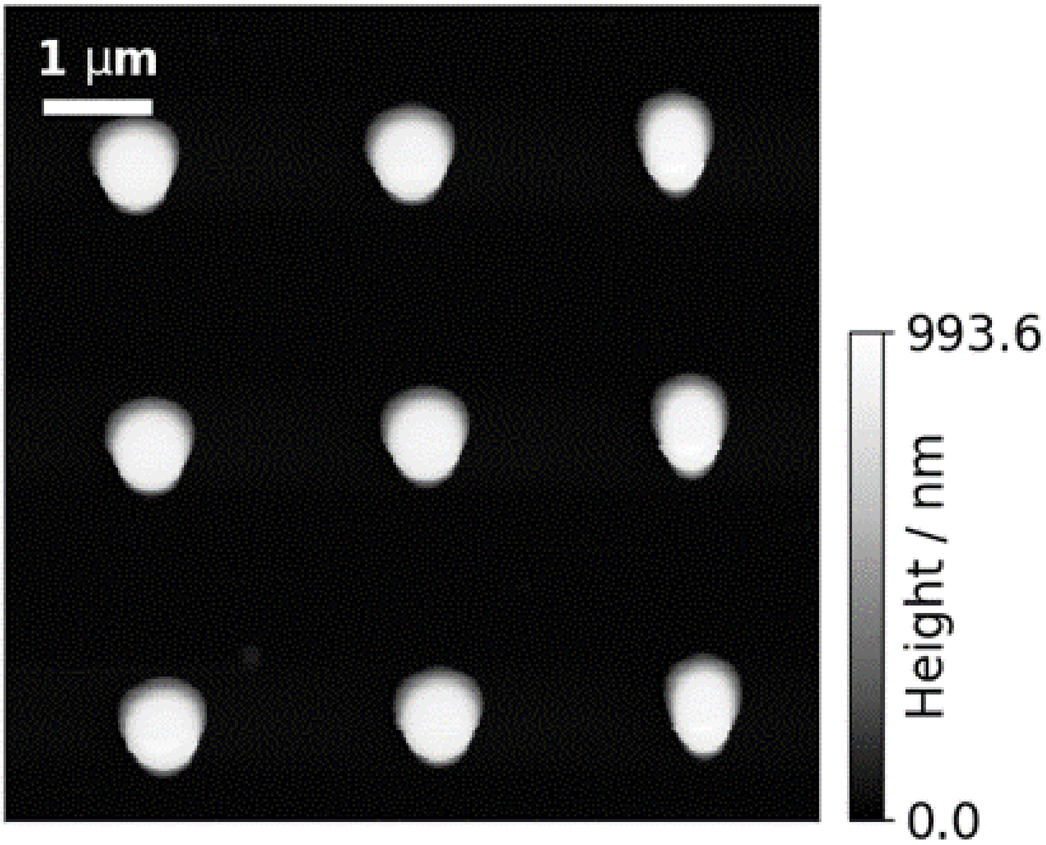

Figure S5: Topography image of the selected pillars on SU-8 only sample.

S4 Finite element method modeling

We employed COMSOL Multiphysics software to model heat transfer and solid mechanics in heterostructures, aiming to understand laser-induced temperature changes and thermal expansion in solids. Our approach involved utilizing a two-dimensional asymmetric model with an extra-fine free triangular mesh, allowing us to analyze heat transfer within the absorber and between different materials such as PMMA, SU-8, air and the silicon substrate. In our thermal expansion model, we coupled the interface of heat transfer in solids and fluids to solid mechanics. The thermal and mechanical boundary conditions were set as illustrated in Figure S6. Adiabatic boundary conditions were applied at the outer boundaries as indicated by the red lines in the heat transfer model and free surface boundary conditions at the PMMA/air interface (purple lines) and fixed constraint boundary conditions at the silicon/air interface (orange lines). The pulse peak laser power was set to 20 mW, the laser spot radius to 20 µm, the laser repetition rate to 710 kHz and the laser pulse width to 220 ns. The width and thickness of the silicon substrate and air geometry were set to 5 µm each.

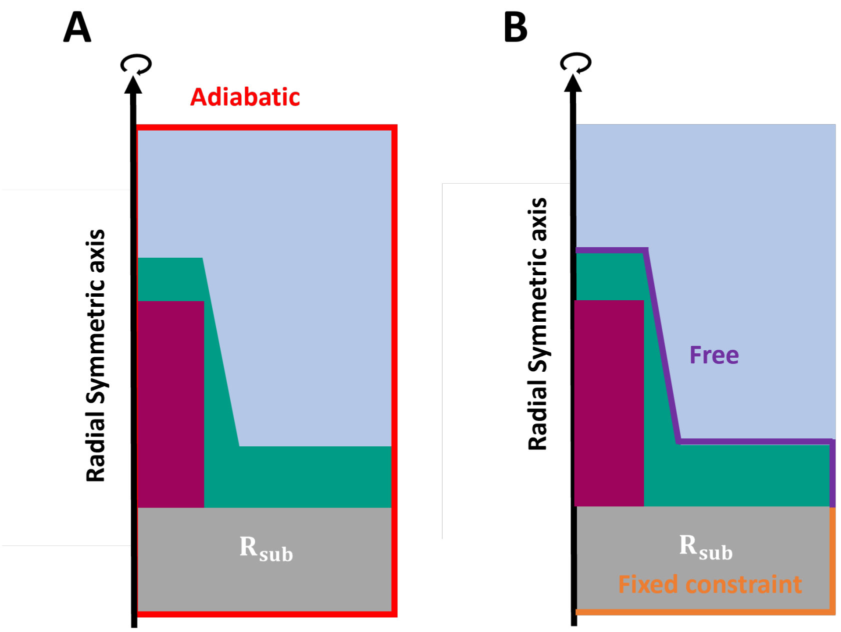

Figure S6: (A) Boundary conditions used to model the photothermal heating in the finite element method simulations, (B) Boundary conditions used in the thermo-elastic simulations.

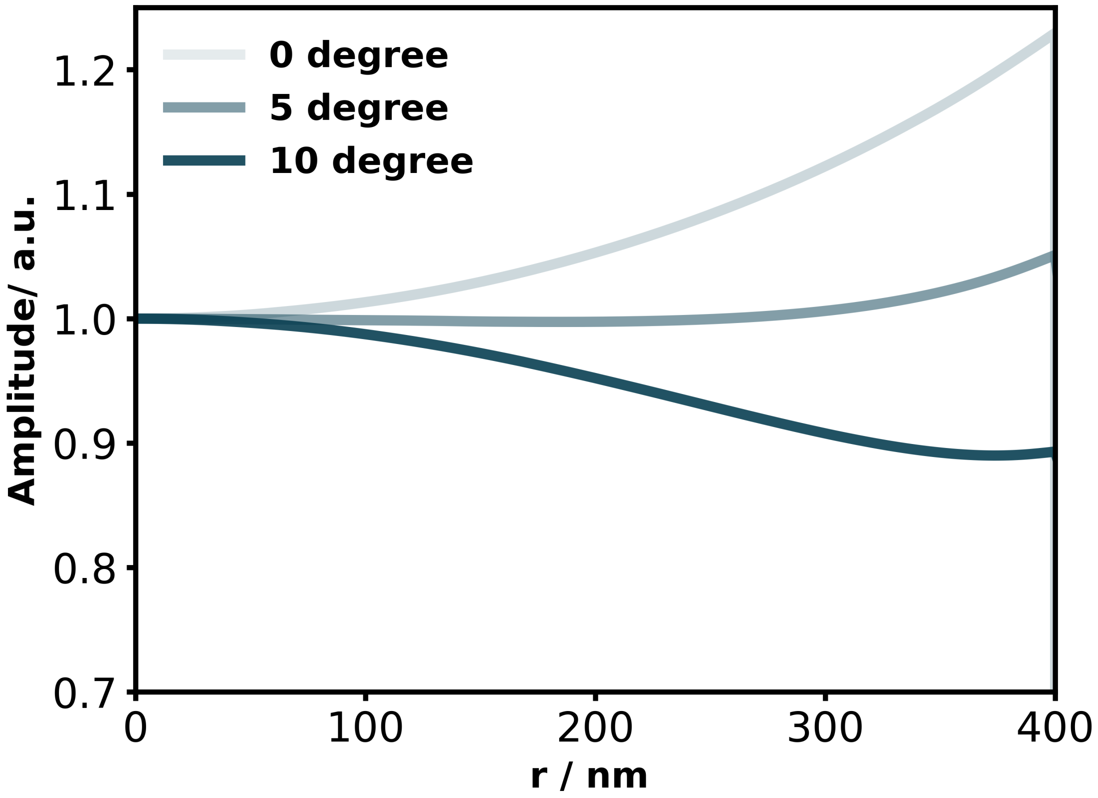

Figure S7: (A) The normalized amplitude of the minimum surface deformation at different radial positions with varying tilted angles of the sidewalls, considering the same thermal expansion coefficient for PMMA and SU-8.

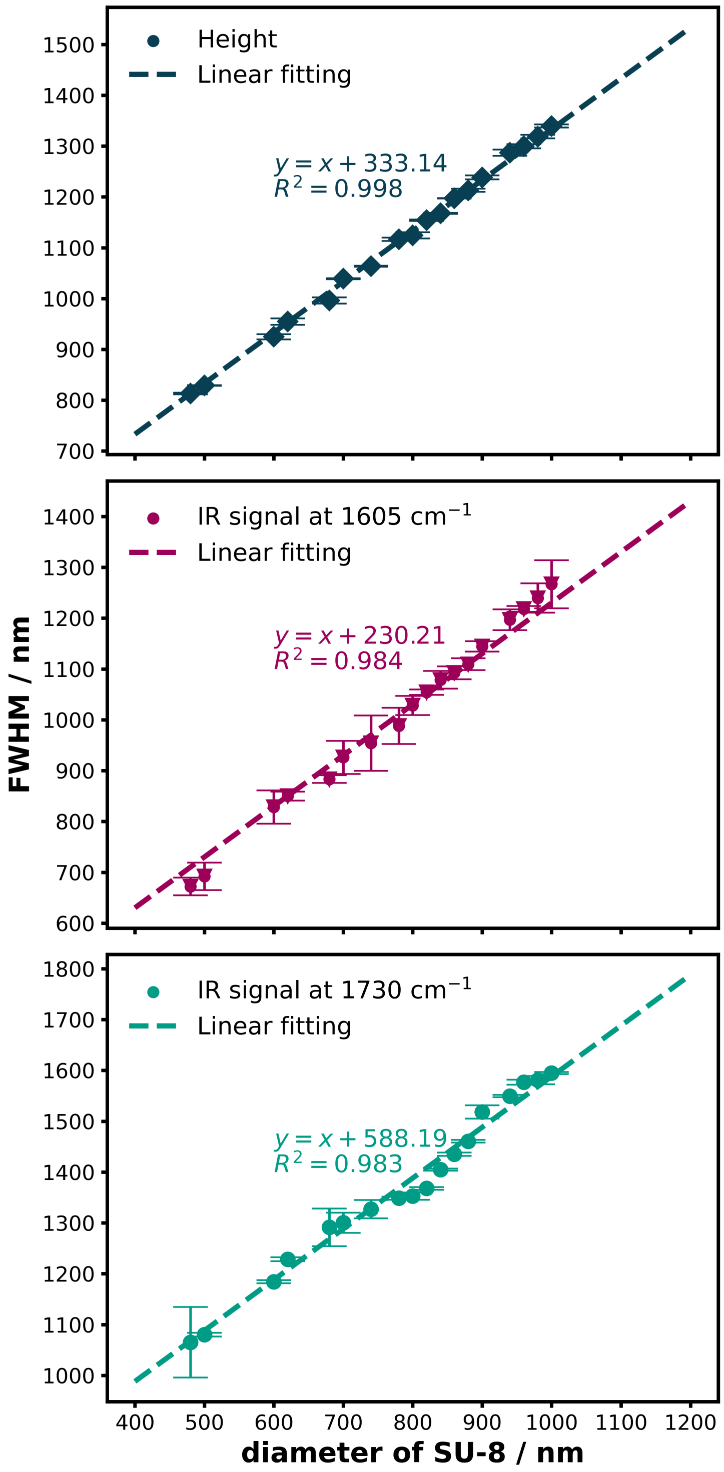

Figure S8: The linear fitting of experimental data was performed using the FWHM values from the cross-sectional profiles of the height image and the chemical images obtained at 1605 cm^{-1} and 1730 cm^{-1}.

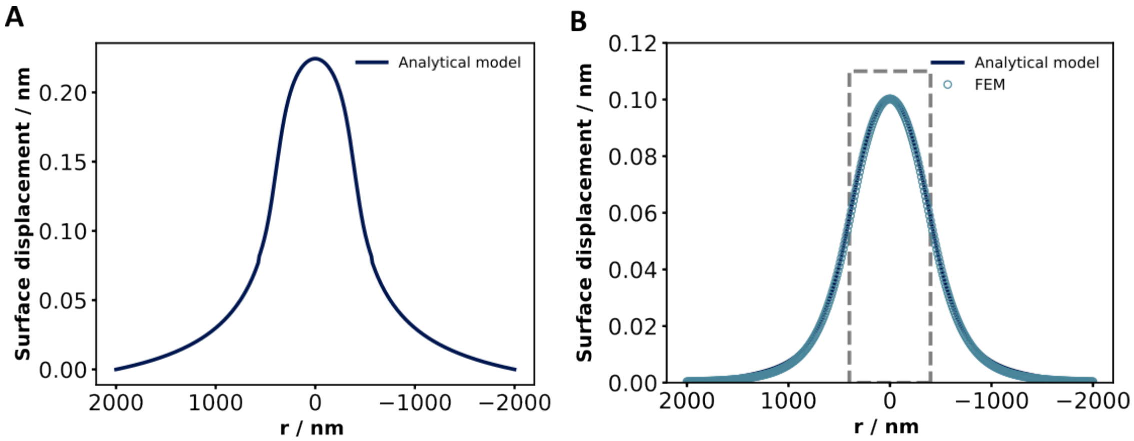

Figure S9: (A) Analytical simulation of the surface displacement for an SU-8 pillar with a radius of 400 nm and vertical side walls. (B) Analytical and FEM simulations were performed to investigate the surface displacement of an SU-8 pillar embedded in a PMMA matrix, where the pillar is flush with the surrounding matrix, and the matrix has a radius of 5 µm.

S5 Code and raw data

Code and raw data to generate all figures is available on Zenodo (DOI: https://doi.org/10.5281/zenodo.14812045)[S20].

References

[S1] Cole, K.; Beck, J.; Haji-Sheikh, A.; Litkouhi, B. *Heat Conduction Using Greens Functions*, 0th ed.; CRC Press, 2010.

[S2] Hahn, D. W.; Özişik, M. N. *Heat conduction*, 3rd ed.; Wiley: Hoboken, NJ, 2012.

[S3] Cole, K. D., Ed. *Heat conduction using Green's functions*, 2nd ed.; Series in Computational and Physical Processes in Mechanics and Thermal Sciences; CRC Press: Boca Raton, 2011.

[S4] Noda, N.; Hetnarski, R. B.; Tanigawa, Y. *Thermal stresses*, 2nd ed.; Taylor & Francis: New York, 2003.

[S5] Faria, L.; Moreira, R. L. Infrared spectroscopic investigation of chain conformations and interactions in P(VDF-TrFE)/PMMA blends. *Journal of Polymer Science Part B: Polymer Physics* **2000**, *38*, 34–40.

[S6] Ripalda, J. M.; Álvaro, R.; Dotor, M. L. Ether cleavage and chemical removal of SU-8. *SciPost Chemistry* **2023**, *2*, 001.

[S7] Wang, J.; Lee, M. K.; Park, S.-M.; Hong, S.; Kim, N. A study on the mechanical properties and deformation behavior of injection molded PMMA-TSP laminated composite. *Korea-Australia Rheology Journal* **2012**, *24*, 23–33.

[S8] Gao, J.; Guan, L.; Chu, J. Determining the Young's modulus of SU-8 negative photoresist through tensile testing for MEMS applications. Hangzhou, China, 2010; p 754464.

[S9] Henins, I. Precision density measurement of silicon. *Journal of Research of the National Bureau of Standards Section A: Physics and Chemistry* **1964**, *68A*, 529.

[S10] Assael, M. J.; Botsios, S.; Gialou, K.; Metaxa, I. N. Thermal Conductivity of Polymethyl Methacrylate (PMMA) and Borosilicate Crown Glass BK7. *International Journal of Thermophysics* **2005**, *26*, 1595–1605.

[S11] Wang, M.; Ramer, G.; Perez-Morelo, D. J.; Pavlidis, G.; Schwartz, J. J.; Yu, L.; Ilic, R.; Aksyuk, V. A.; Centrone, A. High Throughput Nanoimaging of Thermal Conductivity and Interfacial Thermal Conductance. *Nano Letters* **2022**, *22*, 4325–4332.

[S12] Kadoya, K.; Matsunaga, N.; Nagashima, A. Viscosity and Thermal Conductivity of Dry Air in the Gaseous Phase. *Journal of Physical and Chemical Reference Data* **1985**, *14*, 947–970.

[S13] Gaur, U.; Lau, S.-f.; Wunderlich, B. B.; Wunderlich, B. Heat Capacity and Other Thermodynamic Properties of Linear Macromolecules VI. Acrylic Polymers. *Journal of Physical and Chemical Reference Data* **1982**, *11*, 1065–1089.

[S14] Oh, S. H.; Lee, K.-C.; Chun, J.; Kim, M.; Lee, S. S. Micro heat flux sensor using copper electroplating in SU-8 microstructures. *Journal of Micromechanics and Microengineering* **2001**, *11*, 221–225.

[S15] Nogueira, T.; Botan, R.; Wypych, F.; Lona, L. Study of thermal and mechanical properties of PMMA/LDHs nanocomposites obtained by in situ bulk polymerization. *Composites Part A: Applied Science and Manufacturing* **2011**, *42*, 1025–1030.

[S16] Li, K.; Xu, G.; Huang, X.; Xie, Z.; Gong, F. Manufacturing of Micro-Lens Array Using Contactless Micro-Embossing with an EDM-Mold. *Applied Sciences* **2018**, *9*, 85.

[S17] Dellmann, L.; Roth, S.; Beuret, C.; Racine, G.-A.; Lorenz, H.; Despont, M.; Renaud, P.; Vettiger, P.; De Rooij, N. Fabrication process of high aspect ratio elas-

tic structures for piezoelectric motor applications. Proceedings of International Solid State Sensors and Actuators Conference (Transducers '97). Chicago, IL, USA, 1997; pp 641–644.

[S18] Hopcroft, M. A.; Nix, W. D.; Kenny, T. W. What is the Young's Modulus of Silicon? *Journal of Microelectromechanical Systems* **2010**, *19*, 229–238.

[S19] Cherukuri, R.; Lambai, A.; Sukki, L.; Väliaho, J.; Kallio, P.; Sarlin, E.; Ramachandramoorthy, R.; Kanerva, M.; Mohanty, G. In-situ SEM micropillar compression and nanoindentation testing of SU-8 polymer up to 1000 s1 strain rate. *Materials Letters* **2024**, *358*, 135824.

[S20] Zhang, Y.; Yilmaz, U.; Vorobev, A. S.; Iadanza, S.; O'Faolain, L.; Lendl, B.; Ramer, G. Data and Code for "Probing the Depths: Experimental and Simulated Insights into Nanoscale AFM-IR Imaging of Complex Heterogeneous Structures". 2025; `https://doi.org/10.5281/zenodo.14812045`.
